# Biochemical and structural characterizations of thioredoxin reductase selenoproteins of the parasitic filarial nematodes *Brugia malayi* and *Onchocerca volvulus*

**DOI:** 10.1016/j.redox.2022.102278

**Published:** 2022-03-04

**Authors:** Francesca Fata, Radosveta Gencheva, Qing Cheng, Rachel Lullo, Matteo Ardini, Ilaria Silvestri, Federica Gabriele, Rodolfo Ippoliti, Christina A. Bulman, Judy A. Sakanari, David L. Williams, Elias S.J. Arnér, Francesco Angelucci

**Affiliations:** a Dept. of Life, Health and Environmental Sciences, University of L'Aquila, L’Aquila, 67100, Italy; b Division of Biochemistry, Department of Medical Biochemistry and Biophysics, Karolinska Institutet, Stockholm, 17177, Sweden; c Dept. of Microbial Pathogens and Immunity, Rush University Medical Center, Chicago, IL, USA; d Dept. of Pharmaceutical Chemistry, University of California, San Francisco, CA, USA; e Department of Selenoprotein Research, National Institute of Oncology, 1122, Budapest, Hungary

**Keywords:** Thioredoxin reductase, Glutaredoxin, Selenocysteine, X-ray crystallography, Auranofin, Thioredoxin reductase inhibitor

## Abstract

Enzymes in the thiol redox systems of microbial pathogens are promising targets for drug development. In this study we characterized the thioredoxin reductase (TrxR) selenoproteins from *Brugia malayi* and *Onchocerca volvulus*, filarial nematode parasites and causative agents of lymphatic filariasis and onchocerciasis, respectively. The two filarial enzymes showed similar turnover numbers and affinities for different thioredoxin (Trx) proteins, but with a clear preference for the autologous Trx. Human TrxR1 (hTrxR1) had a high and similar specific activity versus the human and filarial Trxs, suggesting that, *in vivo*, hTrxR1 could possibly be the reducing agent of parasite Trxs once they are released into the host. Both filarial TrxRs were efficiently inhibited by auranofin and by a recently described inhibitor of human TrxR1 (TRi-1), but not as efficiently by the alternative compound TRi-2. The enzyme from *B*. *malayi* was structurally characterized also in complex with NADPH and auranofin, producing the first crystallographic structure of a nematode TrxR. The protein represents an unusual fusion of a mammalian-type TrxR protein architecture with an N-terminal glutaredoxin-like (Grx) domain lacking typical Grx motifs. Unlike thioredoxin glutathione reductases (TGRs) found in platyhelminths and mammals, which are also Grx–TrxR domain fusion proteins, the TrxRs from the filarial nematodes lacked glutathione disulfide reductase and Grx activities. The structural determinations revealed that the Grx domain of TrxR from *B*. *malayi* contains a cysteine (C22), conserved in TrxRs from clade IIIc nematodes, that directly interacts with the C-terminal cysteine-selenocysteine motif of the homo-dimeric subunit. Interestingly, despite this finding we found that altering C22 by mutation to serine did not affect enzyme catalysis. Thus, although the function of the Grx domain in these filarial TrxRs remains to be determined, the results obtained provide insights on key properties of this important family of selenoprotein flavoenzymes that are potential drug targets for treatment of filariasis.

## 1 Introduction

Neglected Tropical Diseases (NTDs) are a diverse group of approximately 20 conditions or diseases that occur in the tropical and subtropical regions of the world and cause tremendous disabilities to over a billion people living in mostly impoverished countries [1]. Two major NTDs are lymphatic filariasis (LF) caused by the filarial nematodes *Wuchereria bancrofti*, *Brugia malayi*, and *B*. *timori,* which affect 51.4 million people worldwide [2] and onchocerciasis or “river blindness” caused by *Onchocerca volvulus* which infects 20.9 million people mostly living in sub-Saharan Africa [3]. During a blood meal, infected vectors, which are blood-feeding insects, release infectious third-stage larvae into human hosts where larvae develop into adult worms. For LF, adults worms reside in lymphatic tissues and for onchocerciasis, adult worms live in subcutaneous tissues. Adult worms mate and live for 10–15 years generating millions of young larval microfilariae (MF) that are ingested by the insect vector during its blood meal, thus continuing the parasite’s life cycle and perpetuating the transmission of the infection [4]. Mass Drug Administration (MDA), which represents the main strategy to control both diseases, consists of the administration of the three drugs: ivermectin, diethylcarbamazine with albendazole for LF, and ivermectin for onchocerciasis (WHO, 2020). The three drugs act by killing MF, thus preventing transmission but they do not eliminate the adult worms, which remain alive and continue to reproduce [5]. Due to the severe adverse side-effects of ivermectin that can occur in patients with onchocerciasis who are also co-infected with high numbers of MF from another filarial worm, *Loa loa* [6], MDA is not feasible in many endemic areas. In addition*,* with the threat of drug resistance due to large-scale drug administration, identification of valid drug targets and the discovery of new and specific anti-filarial therapies are critically needed [7,8]. To date, there are no vaccines nor drugs that directly kill the adult stages of the filarial worms (macrofilaricides) that cause lymphatic filariasis or onchocerciasis. The only proven macrofilaricidal drug is the antibiotic doxycycline, which eliminates the intracellular bacterial endosymbiont, *Wolbachia* [9,10]. Since the worms that cause these diseases rely on *Wolbachia* for development, fertility, and survival, *Wolbachia* is considered an excellent target for drug therapies. Doxycycline, however, takes ∼18 months to kill adult worms and cannot be administered to young children nor pregnant women. In addition, doxycycline requires 4–6 weeks of treatment which is not feasible for MDA programs [11].

Another approach to eliminate adult filariae is to directly target the worm’s metabolic pathways. Disruption of the molecular pathways can lead to a reduction in fecundity and embryogenesis in female worms, thereby reducing transmission of microfilariae as well as impairing a worm’s ability to maintain homeostasis. A potential winning strategy to develop novel anti-filarial therapies is targeting the parasites’ thiol-redox pathways [12,13]. For example, parasites are subjected to reactive oxygen species (ROS) generated from their own metabolism and to the oxidative stress imposed by the host's immune response, making the antioxidant system indispensable for worm survival. Proteins implicated in the ROS detoxification mechanisms are promising targets in drug discovery projects aimed at eliminating diseases in general [[14], [15], [16], [17], [18], [19], [20]]. Redox balance in most organisms is controlled by the action of two parallel systems, one based on glutathione (GSH), and the other based on isoforms of thioredoxin (Trx), both of which play central roles in regulating redox homeostasis and are involved in crucial physiological functions such as DNA synthesis [[21], [22], [23]]. In both the GSH and Trx systems, reducing equivalents are typically obtained from NADPH and are channeled to these systems by either glutathione-disulfide reductase (GR) or thioredoxin reductase (TrxR). TrxRs represent promising pharmacological targets to fight both prokaryotic and eukaryotic pathogens [14,24,25]. Nematodes and platyhelminths, the two phyla to which parasitic worms belong, have different thiol-redox pathways. Nematodes possess conventional GR and TrxR [26], while platyhelminths have a single TrxR-like enzyme for reducing both oxidized glutathione (GSSG) and Trx, called thioredoxin glutathione reductase (TGR; [16]). Helminth TrxRs are members of the high molecular weight TrxR subfamily and are selenocysteine (Sec, U)-containing homodimeric flavoenzymes. They transfer reducing equivalents from NADPH to their flavin adenine dinucleotide (FAD) cofactor and, subsequently, to a pair of cysteines located at the *si*-face of the isoalloxazine ring. From there, electrons are transferred to the mobile Sec-containing C-terminal redox center of the symmetrical subunit, that, upon reduction of its Cys-Sec motif, reaches the solvent-exposed surface of the enzyme transferring electrons to the incoming oxidized Trx [27]. Using auranofin, a gold-containing compound, which has been shown to inhibit TrxR and to kill parasites both *in vitro* and in animal models, indicates that TrxR from filarial nematodes is a druggable target [28,29]. TGR are TrxR variants in which a TrxR domain is fused at its N-terminal end with a glutaredoxin (Grx) domain. Grxs are small proteins capable of reducing GSH mixed disulfides and carrying out a wide range of redox functions in cells [21]. Parasite TGRs are crucial for parasite survival and have been found to be promising drug targets in pathogenic platyhelminths, such as *Schistosoma* spp., being the only enzymes that concomitantly support the Trx and GSH pathways [[18], [30]]. Indeed, the fusion of the two domains should confer to the enzyme the ability to reduce oxidized Trx, as well as GSSG, and mixed disulfides (GSSX). The latter two functions require electron transfer from the TrxR domain to the Grx domain [31,32]. This peculiar fusion seems strictly related to parasitic cestodes and trematodes; free-living platyhelminths have separate reductases, thus indicating that canonical reductases were specifically lost in the parasitic lineages [16,33].

In the current work, we present the biochemical analyses of TrxR from *B*. *malayi* (BmTrxR) and *O. volvulus* (OvTrxR). We also solved the crystal structure of BmTrxR, being the first crystallographic structure of a TrxR from a nematode, which, surprisingly, is a fusion of a large TrxR domain with a Grx-like domain at the N-terminus lacking a typical Grx active site motif. This architecture is reminiscent of TGRs from parasitic platyhelminths; however, at odds with this, BmTrxR is not endowed with Grx or GR activities. Preliminary bioinformatic analyses show that this unusual domain fusion is found in parasitic nematodes belonging to clade III subgroup c (Spiruromorpha + others; see Ref. [34], including the OvTrxR (87% sequence identity with BmTrxR) thus indicating a possible role of this unique form of TrxR in the pathogenesis of these filarial parasites. The findings reported herein can serve as a starting point both to understand the role of the additional Grx domain in the nematode parasite life cycle, and to undertake hit-to-lead studies necessary to identify novel preclinical candidates against filarial nematodes.

## 2 Results and discussion

### 2.1 TrxRs and Trxs from *B. malayi* and O. volvulus: sequence comparisons

**Fig. 1:**
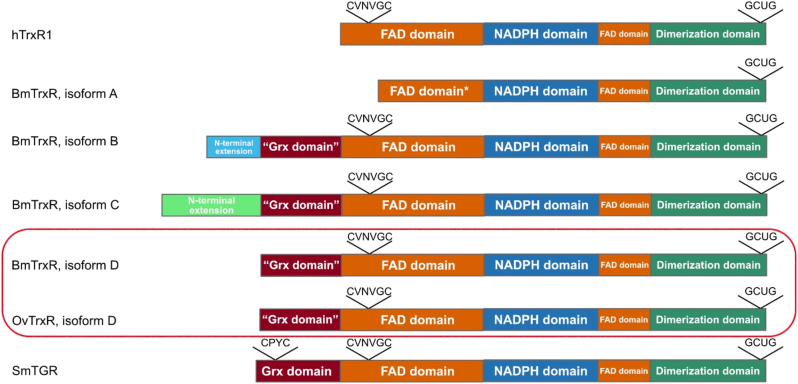
**Domain organization of the selenoprotein TrxR enzymes characterized in this study.** The human TrxR1 (hTrxR1) monomer is shown with a FAD-binding domain (orange) containing a CVNVGC redox active motif, an NADPH binding domain (blue) and the dimerization domains (dark green) guiding association of the two monomers of the dimeric holoenzyme, with the GCUG active site motif at its C-terminus. As shown here, all isoforms of BmTrxR, OvTrxR and SmTGR share this domain organization, albeit with the A isoform of BmTrxR having a truncated and likely non-functional FAD domain, and the remaining forms containing an additional N-terminal Grx domain (burgundy). However, only SmTGR has a typical Grx active site motif (CPYC) in its Grx domain, while that is lacking in the BmTrxR or OvTrxR enzymes. Isoform B of BmTrxR have similar potential secretory signal peptides at their N-termini (light blue, as predicted with TargetP; https://services.healthtech.dtu.dk/service.php?TargetP-2.0) while isoform C of BmTrxR has another form of potential signal peptide at its N-terminus for unknown compartmentalization (light green, as predicted with TargetP). The enzymes studied herein are isoform D of both BmTrxR and the OvTrxR, boxed in red. For an alignment with the full amino acid sequences of the BmTrxR enzymes and indicated degrees of homology, see Supplementary Fig. S3A. (For interpretation of the references to color in this figure legend, the reader is referred to the Web version of this article.)

The BmTrxR and OvTrxR are selenoproteins with a C terminal penultimate Sec within a GCUG active site motif and the classic TrxR redox active site sequence CVNVGC in the FAD domain. Given the presence of a Grx domain (Fig. 1), it was of interest to compare the sequence homology of the filarial TrxR to both human cytosolic TrxR1 (hTrxR1) and TGR from *Schistosoma mansoni* (SmTGR). Both sequence alignments and secondary structure predictions suggest higher similarity of the TrxR domain of filarial nematodes to hTrxR1 than to that of SmTGR. Four different isoforms (A-D) of BmTrxR, which are likely different splicing forms of the same gene, all contain Sec in the penultimate position of the polypeptide (See Supplementary Figs. S1 and S2). Isoforms B, C, and D are characterized by identical TrxR domains (residues 143–638 in isoform B, residues 218–713 in isoform C and residues 103–598 in isoform D) that share 57% sequence identity with hTrxR1 (PDB ID: 3QFA [27]; and *Rattus norvegicus* TrxR (PDB ID 1H6V; [35]. Isoform A is likely not to be produced *in vivo* given that it lacks a crucial portion of the FAD binding site, as also inferred by the 3D homology modelling of its TrxR domain (results not shown). The B, C, and D isoforms have N-terminal extensions of different lengths (B: 142; C: 217; D: 102) in addition to the TrxR module containing an identical Grx domain as well as potential signal peptides or mitochondrial transfer peptides in the B and C forms. The BmTrxR N-terminal extensions were queried against the *O. volvulus* genomic sequence to generate potential OvTrxR alternative splice variants. The results suggest that the same splice variants occur in *O. volvulus* TrxR (not shown). For the present study, we characterized the D isoform of BmTrxR and the corresponding isoform of OvTrxR, sharing 87% sequence identity. The domain organization of the enzymes is schematically shown in Fig. 1, with the full sequence alignment shown in Supplementary Fig. S3.

There are two Trx genes in both *B. malayi* and *O. volvulus,* according to the wormbase parasite database (https://parasite.wormbase.org/Multi/Tools/Blast/Results?tl=WE7S61nvEwVnSpqy-6179428). We refer to these isoforms as BmTrx1, BmTrx2, OvTrx1 and OvTrx2. All four proteins are predicted to have typical Trx-like domains, with a -CXXC- active site predicted to catalyze the reduction of downstream protein substrates. The genes of BmTrx1 and BmTrx2 are placed adjacent on the chromosome. Both genes have the same intron/exon structures with only one different intron at the center of the sequence. They also differ at the canonical disulfide active site: BmTrx1 has a -CPPC- motif while BmTrx2 has a -CPQC- motif. An earlier study suggested that these two variants are allelic forms of BmTrx and not separate genes [36]. We also compared the sequences of the Trx isoforms BmTrx1-2 and OvTrx1-2 with hTrx1. Among the characteristics of hTrx1 is the presence of additional non-catalytic Cys residues capable of forming dimers and intramolecular structural disulfides, which participate in redox signaling regulation [37]. The OvTrx2 isoform may contain one such structural Cys, while BmTrx1/2 and OvTrx1 have only two Cys residues each, constituting the active site dithiol/disulfide motif. A CGPC sequence is typical for Trx active sites, but the filarial Trx isoforms instead present either a CPPC motif (BmTrx1 and OvTrx1) or a highly unusual CPQC sequence (BmTrx2 and OvTrx2). For full sequences and an alignment with hTrx1, see Supplementary Fig. S3B.

### 2.2 *Biochemical characterization of* isoforms D of *BmTrxR and OvTrxR and their comparison with hTrxR1*

**Fig. 2:**
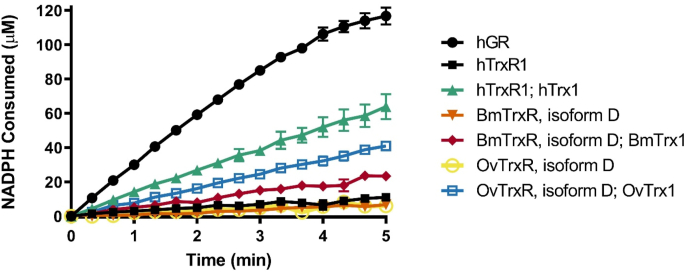
**Glutathione disulfide reduction by hGR, hTrxR1, BmTrxR isoform D or OvTrxR isoform D with or without coupling to Trx.** Absorption at 340 nm was followed in a reaction containing 1 mM GSSG as substrate together with 15 nM of each respective reductase and 0.25 mM NADPH, with or without addition of 25 μM thioredoxin, as indicated. NADPH consumption in the reaction was quantified using a standard curve of pure NADPH. Error bars represent standard deviation of the mean.

BmTrxR and OvTrxR were recombinantly expressed in *Escherichia coli*. BmTrxR and OvTrxR both have typical TrxR absorption signatures confirming the presence of FAD with a 463 nm peak (Supplementaryl. Fig. S4a). Considering that BmTrxR and OvTrxR also carry N-terminal Grx domains, albeit without typical Grx active site dithiol motifs (see Fig. 1 and below), we first assessed whether the two enzymes possessed GSSG reductase (Fig. 2) and Grx activity (Supplementary Fig. S5). Such activities are typically found with TGR enzymes carrying Grx domains [14,16,38]. The resulting analyses showed that the filarial enzymes are devoid of any apparent GSSG reductase or Grx activity. However, since human TrxR1 is devoid of GR activity but can support reduction of GSSG when coupled to Trx1 [39] we also tested such Trx-coupled assays with the filarial enzyme systems. This showed that OvTrxR or BmTrxR together with OvTrx or BmTrx, respectively, could indeed support some GSSG reduction, but not as efficiently as the human enzymes. These results are summarized in Fig. 2.

We next assessed whether BmTrxR and OvTrxR have typical TrxR activities with regards to reduction of the synthetic model substrate DTNB, which is the substrate classically used for definition of units of TrxRs [40]. This analysis revealed that human TrxR1 is clearly more efficient in reducing of DTNB than the filarial enzymes, mainly due to lower *K*_*m*_ but also with a higher k_*cat*_, resulting in about 2- to 3-fold higher k_*cat*_/*K*_*m*_ ratios than the filarial enzymes. It was clear, however, that DTNB was still a rather efficient substrate for all three enzymes in terms of maximal turnover, k_*cat*_ (Table 1). It should be noted that absolute turnover with these enzymes will also be dependent upon their Sec contents, but with the enzymes produced recombinantly under identical conditions (see *Methods*), we trust that the respective comparisons in kinetic parameters as performed here will be illustrative comparisons with relevance also for the native enzymes.

**Table 1:** Kinetic parameters of BmTrxR, OvTrxR and hTrxR1 with DTNB, hTrx1, BmTrx1, BmTrx2, BmTrxP40G, OvTrx1, and OvTrx2, calculated based on activity data from Fig. 3.

Enzyme	Substrate	Active site	K*m* (μM)	k_*cat*_ (min^−1^)	k_*cat*_*/*K*m* (min^−1^ μM^−1^)
BmTrxR	DTNB		70 ± 7.8	1200 ± 57	17
	hTrx1	-CGPC-	21 ± 3.7	430 ± 46	20
	BmTrx1	-CPPC-	2.7 ± 1.4	380 ± 25	140
	BmTrx2	-CPQC-	4.7 ± 0.6	680 ± 30	140
	BmTrx P40G	-CGPC-	4.7 ± 0.9	570 ± 39	120
	OvTrx1	-CPPC-	1.1 ± 0.3	199 ± 13	180
	OvTrx2	-CPQC-	9.6 ± 3.5	48 ± 6.9	5.0
OvTrxR	DTNB		170 ± 12	1700 ± 34	10
	hTrx1	-CGPC-	14 ± 1.4	720 ± 39	52
	BmTrx1	-CPPC-	2.4 ± 1.2	440 ± 43	180
	OvTrx1	-CPPC-	1.6 ± 0.4	301 ± 19	190
	OvTrx2	-CPQC-	4.5 ± 2.2	50 ± 6.4	11
hTrxR1	DTNB		61 ± 8.4	2500 ± 120	42
	hTrx1	-CGPC-	4.3 ± 1.2	1040 ± 79	242
	BmTrx1	-CPPC-	4.4 ± 1.1	860 ± 59	200
	OvTrx1	-CPPC-	6.1 ± 2.1	960 ± 110	157
	OvTrx2	-CPQC-	8.5 ± 4.1	91 ± 16	11

**Fig. 3:**
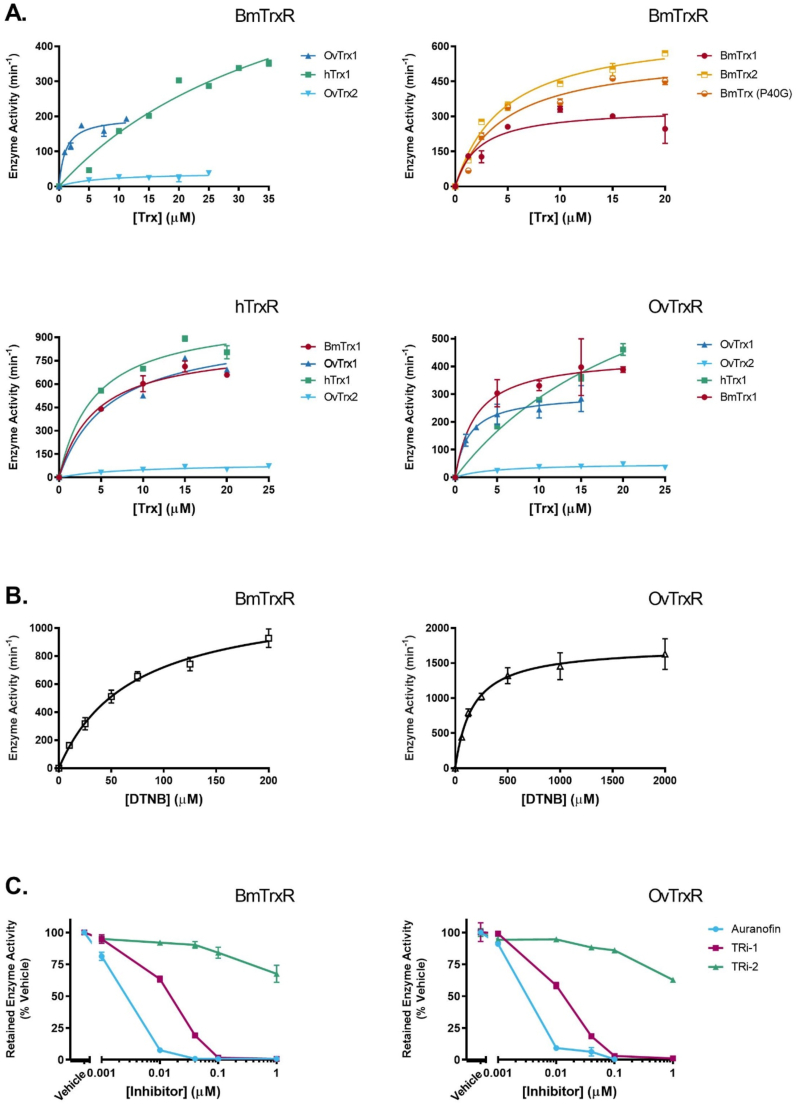
**Enzyme activity of BmTrxR (isoform D), OvTrxR (isoform D) and hTrxR with substrates (A**–**B) and inhibitors (C).** A: Enzyme activities with BmTrx1, BmTrx2, mutant BmTrx (P40G), OvTrx1 and hTrx1 were measured with 0.25 mM NADPH, 0.16 mM insulin, and 20 nM TrxR (except for 40 nM BmTrxR with OvTrx1), by following A_340_ and using a NADPH standard curve. Enzyme activities with OvTrx2 were measured with 0.25 mM NADPH, 0.16 mM insulin and 80 nM of TrxR, by following A_340_ and using a NADPH standard curve. B: Enzyme activities with DTNB were measured with 10 nM OvTrxR or 20 nM BmTrxR and 0.25 mM NADPH, by following A_412_ for 5 min and using εA_412_ = 13,600 M^−1^cm^−1^. C: Inhibition of 1 mM DTNB reduction by auranofin, TRi-1 or TRi-2, when incubated with 25 nM OvTrxR or BmTrxR for 30 min with 0.25 mM NADPH and 0.1 mg/ml BSA. Relative enzyme activity was assessed by following A_412_ upon addition of DTNB and normalized to controls incubated with 1% v/v DMSO (vehicle). Error bars represent standard deviation of the mean.

The reduction of different Trx isoforms by the filarial enzymes was subsequently assessed, using the classical Trx-linked insulin disulfide reduction assay [40,41]. Firstly, all Trx isoforms were confirmed to be able to support insulin reduction when coupled to DTT, but not when coupled to GSH (showing that the proteins do not have typical GSH-dependent Grx activities); BmTrx and OvTrx1 showed similar and efficient DTT-mediated insulin reduction, while OvTrx2 was the least efficient Trx protein of the three (Supplementary Fig. S4b), in accordance with the results of the DTNB assay reported in Table 1 and Fig. 3.

Both filarial TrxR enzymes were able to use hTrx1 as substrate and displaying similar kinetic parameters, although BmTrxR was slightly more efficient in this assay than OvTrxR. Neither of the two enzymes were, however, as efficient in reducing hTrx1 as the cognate hTrxR1. Conversely, BmTrxR and OvTrxR were clearly more efficient in reducing their cognate Trx proteins (Table 1). Varying an active site proline of the CPPC motif of BmTrx1, obtaining BmTrx2 (CPQC), or mimicking the active site motif of hTrx1 (CGPC) did not have any significant effect on the k_*cat*_/*K*_*m*_ ratios with BmTrxR but gave slightly lower affinity and higher maximal turnover. Since BmTrxR has a much lower affinity for hTrx1 than for the BmTrx P40G mutant, the first proline of the active site sequence of the thioredoxin appears to not be the determining factor in BmTrxR’s binding affinity. Mutating both active site cysteine residues in BmTrx1 resulted in complete abrogation of BmTrxR-coupled activity (not shown), confirming a reaction mechanism mimicking that of hTrxR with hTrx [42]. Interestingly, however, neither of the three flavoenzyme variants showed much activity with the second thioredoxin found in *O*. *volvulus,* OvTrx2 (Table 1), which also harbors an unusual active site motif; this suggests that OvTrx2 may have a different, specialized function in *O*. *volvulus.*

The curves of the assays described above and with kinetic parameters summarized in Table 1 are shown in Fig. 3. Overall, these results clearly demonstrate that each of the TrxR isoforms have, not surprisingly, evolved to be the most efficient reductases together with their main cognate Trx proteins (from the same species). It is, however, interesting to note the rather high activity with the filarial thioredoxins displayed by hTrxR1, thus suggesting that the human enzyme might contribute to OvTrx1 or BmTrx reduction during infections, should the two enzymes ever be localized to the same compartment. Reports of filarial Trx being secreted during multiple life stages in *B. malayi* and the effects of BmTrx on mammalian cell p38 MAPK signaling [36] make a case for the possibility for clinically significant interactions between hTrxR1 and filarial Trxs *in vivo*.

### 2.3 Inhibition of nematode TrxRs by auranofin, TRi-1 and TRi-2

In addition to displaying similar kinetic profiles, BmTrxR and OvTrxR also had similar sensitivities to inhibition by well-characterized human TrxR inhibitors. As expected, auranofin, a pan-TrxR inhibitor [[43], [44], [45], [46]], also capable of targeting SmTGR [47], was here found to efficiently inhibit also BmTrxR and OvTrxR (Fig. 3C). The novel thioredoxin reductase inhibitors TRi-1 and TRi-2 were optimized for selective hTrxR1 but not GR inhibition, with TRi-1 being most efficient at inhibiting cytosolic TrxR1 over mitochondrial TrxR2 [45]. TRi-1 was recently also found to be significantly more specific in TrxR1-targeting than auranofin within a cellular context [48]. Here we found that the apparent IC_50_ with both BmTrxR and OvTrxR was similar to that with hTrxR1 (Table 2, Fig. 3C). Given the high efficacy of auranofin treatment in reducing worm burdens [28], the efficient inhibition of BmTrxR and OvTrxR by TRi-1 might have similar antifilarial effects, and thus TRi-1 could be evaluated for antifilarial activity.

**Table 2:** Inhibition parameters of 25 nM BmTrxR (isoform D), 25 nM OvTrxR (isoform D) and 15 nM mammalian TrxR1 with auranofin, TRi-1 and TRi-2, based on data from Fig. 3C.

Inhibitor	Enzyme	Apparent IC_50_ (μM)^a^
Auranofin	BmTrxR	0.0024
	OvTrxR	0.0032
TRi-1	BmTrxR	0.013
	OvTrxR	0.012
	Mammalian TrxR1	0.012^b^
TRi-2	BmTrxR	1.8
	OvTrxR	1.5
	Mammalian TrxR1	2.144^b^

a Based on enzyme activity in DTNB reduction assay.

b Data from [45].

### 2.4 Structural characterization of BmTrxR

**Fig. 4:**
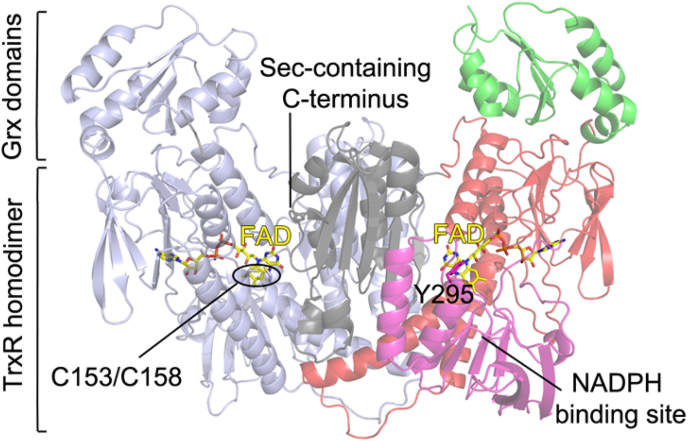
Cartoon representation of the crystallographic structure of TrxR from *B*. *malayi*. One subunit of the homodimer is colored in light blue, while the other is colored accordingly to the different protein domains. The BmTrxR subunit can be divided into four typical subdomains: the Grx domain (1–102, in green), the FAD binding site at the N-terminus (103–259 and 396–465, in red), the central NADPH binding site (260–395, in magenta) and a C-terminal domain at the interface of the two subunits (466–598, in grey). The overall architecture of the border area between the NADPH binding site and the FAD redox site, characterized by the isoalloxazine ring of the FAD (in yellow sticks), the Cys pair at its si-face (C153–C158, in light blue sticks on the left subunit), and the rotating tyrosine (Y295, in magenta sticks on the right subunit), known to change conformation upon NADPH entry [52], is maintained with respect to the other structurally characterized members of the high molecular weight TrxR subfamily. (For interpretation of the references to color in this figure legend, the reader is referred to the Web version of this article.)

Expressing isoform D of BmTrxR, containing the shortest N-terminal extension with respect to the TrxR and Grx domains that are identical in all three isoforms (Fig. 1) and utilizing a more recent method for Sec insertion capable of yielding 95–100% of the wild-type form [49], resulted in a protein producing crystals diffracting up to ∼2.5 Å in C212121 space group. After molecular replacement using as a search model human TrxR isoform I (pdb ID: 3QFA), each TrxR domain was characterized by an additional, large electron density at their N-termini. A modest sequence homology of 28% (over 78 residues pairwise aligned) between the first 102 residues of BmTrxR and the Grx2 from *Clostridium oremlandii* (PDB ID: 4TR1) [50]; suggested that the elongation at the N-terminus may be characterized by a Grx fold, even though none of the canonical Grx active sites belonging to monothiol or dithiol Grxs are present in the N-terminal extension of the TrxR domain. The protein structure has been built starting from residue 14 to 587 and from residue 595 to 598 at the C-terminal end of the protein (Fig. 4). Upon model building, BmTrxR appears to be a fusion of a Grx-like domain (14–102) with a TrxR domain (103–598), reminiscent of the W-shape architecture found in TGR from platyhelminths [51] (Fig. 4). Protein dimerization takes place through the TrxR domain, while the Grx domains lie in the upper external arms of the “W”. There are 3 protein subunits in the asymmetric unit of the crystal lattice; 2 of the 3 subunits, subunit a and b, belong to a physiological dimer while the third, subunit c, forms a physiological dimer with a symmetry-related subunit belonging to an adjacent unit cell. Subunit c is not characterized by continuous and clear electron density especially in some parts of the Grx domain, likely due to an increased mobility in the crystal lattice as indicated by its higher averaged B-factor (83.3 Å^2^ for subunit a; 91.1 Å^2^ for subunit b; 130.9 Å^2^ for subunit c). Therefore, analysis of the structure relies only on the a/b physiological dimer. In this manuscript, we report three different crystal structures: BmTrxR in apoform (2.55 Å; PBD ID: 7P0X), BmTrxR in complex with NADPH (2.8 Å; PDB ID: 7PUT), and BmTrxR in complex with the gold atom released by the drug auranofin (3.1 Å; PDB ID: 7PVJ).

### 2.5 The TrxR domain

Each BmTrxR subunit preserves the overall fold of the other members of the high molecular weight TrxR subfamily (see Ref. [35] and Fig. 1).

**Fig. 5:**
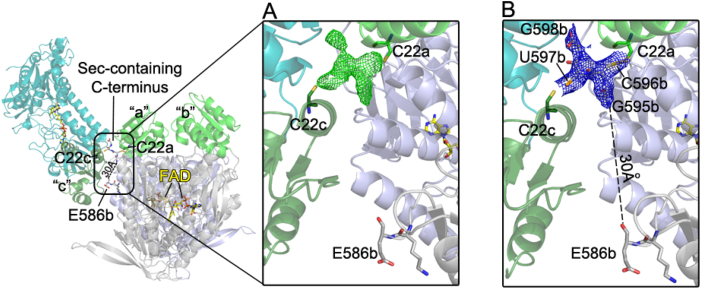
On the left, subunit orientation in the asymmetric unit of the crystal lattice of BmTrxR in the C2221 space group. The TrxR domain of subunit c is in deep teal while the TrxR domains of the a/b physiological dimer are in grey and light blue. The Grx domain of subunit c is in deep green while the Grxs of the a/b dimer are both in green. C22 in the Grx domain of subunit a (C22a), belonging to the a/b physiological dimer, faces C22 of subunit c (C22c) of an adjacent unit cell; the C-terminus of subunit “b” is caught between these two cysteines. In the magnifications on the right, in A, the Fo-Fc difference electron density map (in green meshes) contoured at 3σ indicates the presence of a ligand simultaneously linked to C22a and C22c and, in B, the 2Fo-Fc contoured at 1σ (in blue meshes) with the last residues of the C-terminus fitted (GCUC) is shown. E586b is 30 Å apart from the G595b (heteroatoms are in CPK colors). (For interpretation of the references to color in this figure legend, the reader is referred to the Web version of this article.)

Unusually for this class of proteins [[53], [54]], the last four residues of the Sec-containing C-terminus are here visible in the electron density, thanks to a particular arrangement of BmTrxR subunits in the crystal lattice. The last four residues (-GCUG) of subunit b have been detected in the X-ray derived electron density because they are trapped between subunit a and subunit c belonging to an adjacent unit cell by a disulfide bridge (2.05 Å) between C596(b)-C22(a) and a selenenylsulfide bond (2.1 Å) between U597(b)-C22(c), respectively (Fig. 5). We collected several X-ray data at the Se peak to confirm atom location in crystals of BmTrxR isoform D; however, the anomalous contributions were negligibly low in all the data sets we collected, which is not surprising for a 75-kDa protein with only one Se atom located in a mobile part of the protein as the high B-factors of the Grx domains indicate. Accordingly, the same procedure failed also in the case of human TrxR1 [55], further confirming the difficulty to detect Sec location by anomalous signal in the high molecular weight TrxR subfamily. The residues between E586and G595 are not visible in the electron density. This region of the protein, tethered on one side by the two bridges and on the other side by a salt-link between K494 and E586, includes two glycines (G587 and G595). Because glycines are less constrained in movement with respect to other amino acids due to the absence of the Cβ atom [56], this region may move as a rotating rope making the peptide invisible in the electron density. In support of this view, a 3D model, in which the 8 C-terminal residues missing in the BmTrxR crystal structure were added and geometrically optimized, demonstrates that the space gap between E586 and G595 can be filled by the missing residues (Supplementaryl. Fig. S6). This result suggests the existence of redox wiring also in BmTrxR, between the Grx-like domain, by means of its C22, and the C-terminus of the TrxR domain, a situation reminiscent of TGR from platyhelminths (Fig. 5; [16,[31], [32]]).

### 2.6 The Grx domain

The Grx domain of BmTrxR preserves the common Trx-fold, characterized by a four-stranded β-sheet flanked by four α-helices [57,58]; Supplementaryl. Fig. S7). However, the Grx domain in BmTrxR has an additional two-turns α-helix necessary to join it to the TrxR domain.

This domain does not preserve the classical active site motifs of dithiol or monothiol Grxs [59]. Three cysteines are found in this domain, C22, C82 and C83. While the last two residues are buried, C22 is exposed toward the TrxR domain, about 30 Å apart from the last visible residue of the C-terminus of the TrxR domain of the parent subunit [C22a(CA) - E586b(CA) = 29 Å]. C22 is likely to be a low pK_a_ Cys; it localizes just at the top of an α-helix belonging to the TrxR domain of the same subunit making polar interactions with the carbonyls of F456 and L455 at the top of this helix (the sulfur atom is at 3.4 Å and 3.8 Å from the two oxygens, respectively) and it is within 6.0 Å of the positively charged nitrogens of Arg251 and Lys130 side chains. All these interactions are potentially capable of lowering cysteine pK_a_s in proteins [60]. C22 in each of the three subunits of the asymmetric unit always bears additional electron density on its sulfur. While the strong ramified electron density that initiates from C22 of subunit “a” and extends to C22 belonging to subunit “c” of an adjacent unit cell of the crystal lattice has been interpreted with the C-terminus of subunit “b” trapped between their sulfur atoms (Fig. 5), the additional electron density of C22b is less clear, likely due to a low occupancy of an unrecognized ligand.

In support of the fact that the electron density between the Grx domains of “a” and “b” subunits is dependent on the presence of C-termini forming S–S and Se–S bridges, the same electron density is not detected in both the crystal structures of the BmTrxR isoform B characterized by a selenocysteine content of about 15–20% and of the C22S mutant (Supplementary Fig. S8). The open reading frame of the B isoform of BmTrxR was expressed in *Escherichia coli* with a His-tag at its N-terminus in the presence of pSUABC plasmid as reported previously [28]. In these conditions misreading of the Sec codon (UGA) results in premature termination of the peptide yielding a mixture of 15–20% of the wild-type protein and 80–90% of the truncated form which lacks the last two amino acids [61]. This heterogenous protein mixture yielded crystals diffracting at 3.0 Å in C212121 space group. Thus, C22 is likely a functional cysteine, considering its reactivity and its structural environment, as shown by BmTrxR crystal structures, and earlier extensive bioinformatic analyses that have shown that, in general, Cys exposed on protein surfaces is very limited unless it has function [62,63].

**Fig. 6:**
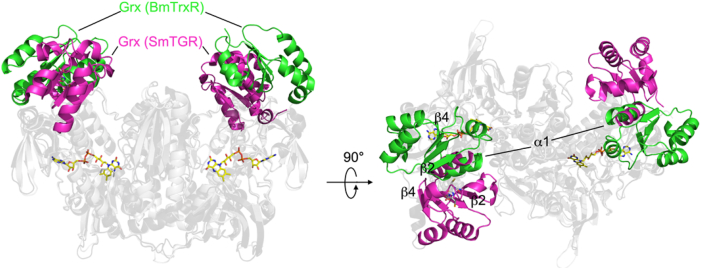
Superposition between the TrxR domains of SmTGR and BmTrxR shown in transparency in black and grey cartoons, respectively, highlighting the different orientation of their relative Grx domains. The Grxs domains of BmTrx are in green while those of SmTGR are in magenta. On the left, it is possible to observe that the Grx domain of SmTGR has rolled over the TrxR domain, using α1 as a pivot, with respect to the Grx domain of BmTrxR. This results in a 180° rotation of the four-stranded β-sheet characteristic of the Grx fold (see Fig. S3). (For interpretation of the references to color in this figure legend, the reader is referred to the Web version of this article.)

The Grx domain contacts the TrxR domain through the following secondary structure elements: α1, α2, β4, α4 and α5 (Supplementary Fig. S7). Their interaction is stabilized by a cation-π interaction (Trp70-Arg417: 3.6 Å), canonical polar interactions [(Glu66(O)-Arg418(NH2):3.3 Å; Gly69(O)-Arg317(NE): 2.8 Å; Lys79 (NZ)-Asp250(OD1): 2.7 Å; Lys79(O)-Arg271(NH2): 2.5 Å; Asn81(ND2)-Tyr428(OH): 2.7 Å] and by several hydrophobic contacts. The other structurally and functionally characterized Grx-TrxR fusion is TGR from platyhelminths and especially that from *S. mansoni* (SmTGR) [[16], [31], [51],^64^,^65^]. This chimeric protein possesses a Grx domain characterized by a canonical dithiol motif (CXXC) and is endowed with Grx and GSSG activity, contrary to what is observed in BmTrxR. Significant sequence identity between the Grx domains of BmTrxR and of TGR from platyhelminths has not been found (using the BLAST algorithm and as a threshold an E-value ≤ 10), reflecting the different modality of interaction with their respective TrxR domains (Fig. 6). In comparison with BmTrxR, the Grx domain of SmTGR has rolled over onto the TrxR domain, using α1 as a pivot, resulting in a rotation of the β-sheet of 180°, making interactions with its TrxR domain mainly with its α1 and β2 structural elements (Fig. 6). The different modality of interaction between the two Grx domains and their respective TrxR domains is also reflected by a different contact surface between the two domains: 897 Å^2^ in BmTrxR and 648 Å^2^ in SmTGR.

BmTrxR is devoid of GR activity (see Fig. 2) and using the HEDS assay, also devoid of Grx activity (Supplementary Fig. S5 [66]); at odds with platyhelminth TGRs. Several monothiol Grxs were found to be inactive in the classical HEDS assay, whose limits have been outlined by Begas et al. [67]. It has recently been found that even if several monothiol Grxs are inactive in HEDS assay, they were found to be active in reducing protein disulfides of several physiologically important proteins; however, for such activity GSH binding and activation is required [22,68]. Intriguingly, a monothiol Grx has been shown to be a substrate of TrxR in *E. coli*, supporting the idea of a possible function of the Grx-TrxR fusions other than GR and Grx activities [69]. Moreover, inactive monothiol Grxs have found to be implicated in iron metabolism [22]. The Grx domain of BmTrxR is devoid of the most important structural features required for GSH binding and activation [70]: it does not have cysteines at the end of α2, where active cysteines of canonical monothiol and dithiol Grxs lie; it is devoid of a crucial glutamine residue on α3 and of a basic residue at the end of β1, and it has the WP motif between α3 and β3 known to decelerate the glutathionylation in inactive Grxs (see Supplementary Fig. S7). In the crystal structure of BmTrxR, C22 is found to interact with the C-terminal arm of the parent subunit. Thus, we speculate that the Grx domain of BmTrxR may potentially work as a disulfide isomerase, as found for some monothiol Grxs, towards specific but still unknown macromolecular substrates present in Clade IIIc nematodes, using as reducing agent the C-terminal arm of the fused TrxR domain. This could potentially also be valid for activities in oxidizing environments, such as the endoplasmic reticulum, considering that isoforms of these TrxR proteins also seem to have signal peptides for secetion (Fig. 1). These possibilities should be further studied.

### 2.7 The NADP(H)-BmTrxR complex

**Fig. 7:**
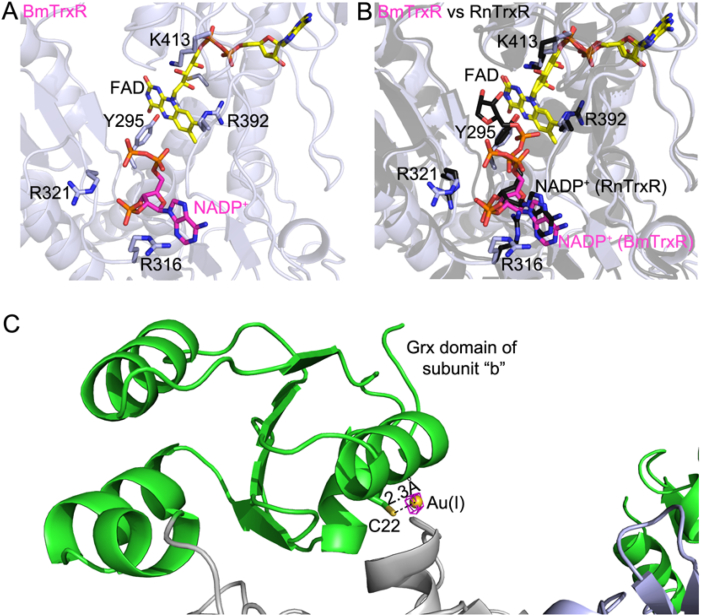
BmTrxR in complex with NADP^+^ and Au(I) from auranofin. In panel A, the NADP(H) binding site of BmTrxR is shown. The BmTrxR structure is colored in light blue, FAD and the bound NADP^+^ are in yellow and magenta sticks, respectively, while the conserved residues important for binding of the reductant are in light blue sticks (heteroatoms are in CPK colors). In panel B, the superposition of BmTrxR and TrxR from *Rattus norvegicus* in complex with NADP^+^ is shown (RnTrxR; PDB ID: 1h6v) [35]. RnTrxR is in black and bound NADP^+^ is in black sticks, as are the conserved residues interacting with it (numbering is according to the BmTrxR). In this case, the ribose bound to nicotinamide is also visible. In panel C, the BmTrxR-gold complex is shown. The anomalous difference Fourier map contoured at 7σ is shown in magenta meshes. The gold atom, shown as yellow sphere, is at 2.3 Å from the sulfur of C22 and its localization superposes with the anomalous Fourier maps. (For interpretation of the references to color in this figure legend, the reader is referred to the Web version of this article.)

The apo crystals of BmTrxR were soaked for 72 h with 500 μM NADPH and the resulting structure was solved at a resolution of 2.8 Å. Only a portion of NADP(H), the 2’-monophosphoadenosine-5’-diphosphate bound to the “a” subunit of BmTrxR at the *re*-face of FAD, was visible in the electron density (Fig. 7, panel A). This moiety of the reductant occupies the same position usually occupied by the analogous atoms of NADP(H) in the crystal structures of other pyridine nucleotide disulfide reductases in complex with NADP(H) (Fig. 7, panel B). In BmTrxR, the β-phosphate of the nucleotide points towards the solvent, suggesting that the remaining part of the reductant, the nicotinamide-ribose moiety, is not detected due its high mobility likely caused by its oxidation. This latter scenario is possible because NADPH after 72 h of soaking into BmTrxR crystals can undergo spontaneous oxidation, yielding NADP^+^, due to the inherent reactivity of reduced FAD towards oxygen [71]. Furthermore, as reported for other homologous flavo-reductases, two positions for the nicotinamide ring exist depending on the redox status of the NADP(H): one relative to the reduced form, where, after rotation of the conserved tyrosine residue, the nicotinamide ring lies parallel to the isoalloxazine ring of FAD to allow electron transfer [31] and the other assigned to NADP^+^, in which the nicotinamide-ribose moiety is flexible and often not visible in the electron density [35,[55], [72], [73]].

### 2.8 The BmTrxR-Auranofin complex

Auranofin is a gold-containing compound in which the Au(I) is tethered between a thioglucose and a triethylphosphine with a linear geometry. It is a well-known selenocysteine-cysteine interacting compound [[47], [74]]. It has already been found to be an inhibitor of BmTrxR *in vitro* and to be effective in reducing *B*. *pahangi* adult worm burdens in animal models [28], and further validated here (Table 2 and Fig. 3C). Crystals of BmTrxR, were soaked with 500 μM NADPH and 4–5 equivalents of auranofin for 72 h, and the resulting structure was solved by molecular replacement at 3.1 Å. After the refinement process and geometry optimization of the model, an anomalous difference Fourier map was calculated from 40 to 3.1 Å and contoured at 7 σ. Its superimposition on the difference density map (Fo - Fc) contoured at the same σ and calculated on the refined BmTrxR structure without the metal, clearly shows the position of the gold atoms in the asymmetric unit (Fig. 7, panel C); an electron density associated with the anomalous signal of a gold atom is present on C22 of subunit b. The gold atom is found at the expected distance for a Au–S bond (2.3 Å) with additional electron density detected from the other side of Au(I) with respect to C22. However, the low resolution of the structure and the incomplete occupancy of the metal (∼50%) prevented identification of the ligand. Crystals derived from co-crystallizations of BmTrxR with stoichiometric equivalents of auranofin in presence of NADPH diffracted around 4 Å but gold was not detected in the electron density maps.

Two scenarios can be proposed to explain the observations when auranofin and NADPH are soaked into BmTrxR crystals: (i) a direct interaction between auranofin and C22 that results in the displacement of thioglucose or phosphine, a view supported by the proposed reactivity of C22 (Fig. 7) or (ii) auranofin primarily interacts with the Sec-containing C-terminus of subunit “a” (being the C-terminus of subunit “b” blocked between the Grx domains of “a” and “c” subunit), where ligands of Au(I) are completely or partially removed by the nucleophilic action of Sec and the proximal Cys, and then, due to the C-terminal movements, transferred to other nucleophilic and interacting sites of the protein, such as C22b. This latter hypothesis is supported by two observations that (i) as demonstrated previously for TrxR and TGR, the gold atom of auranofin targets preferentially the Sec and can be transferred to other nucleophilic sites of the protein [47][74]; and (ii) C22 can interact with the C-terminus (Fig. 7). A structural determination of the enzyme(s) in complex with TRi-1, considering the efficiency in its inhibition (Table 2), would be of interest to solve in future studies.

### 2.9 Functional studies on the role of C22 in BmTrxR

To better understand the potential role for C22 in BmTrxR, the BmTrxR C22S mutant was generated and characterized. Its K_*M*_s measured varying NADPH or the oxidizing substrates (DTNB), the relative k_cat_s and the apparent IC_50_ for auranofin were not statistically different to the relative values determined for the WT enzyme (not shown). Furthermore, the ThermoFAD [75] assay was used to examine the stability of the BmTrxR C22S mutant with respect to the WT [76]. The average melting temperature (Tm) of the wild-type and C22S proteins were 64.5 °C. With the addition of 1 mM NADPH, the average Tm of the wild-type protein was 48.3 ± 0.6 °C and was 49.0 ± 0.5 °C for the C22S mutant (Supplementary. Fig. S9). We conclude that the C22S mutation had no significant effects in classical TrxR assays or in the overall stability of the protein.

### 2.10 Bioinformatic analysis and homology modelling of nematode TrxRs

Analysis of TrxR sequences from nematodes shows that the TrxR domains are highly conserved across all clades while the N-terminal extensions from the same clade are highly conserved but are poorly conserved between clades (Supplementary. Figs. S10 and S11).

Sequence alignment followed by 3D modelling using the BmTrxR structure as a template, revealed that TrxRs in the parasitic nematodes belonging to clade IIIc also possess a Grx-like domain with conservation of the three cysteines and have sequence identities ranging from 62% of *Thelazia callipaeda* to 97% of *B. pahangi* compared to the Grx domain of BmTrxR. The exception is the TrxR of *Dracunculus medinensis* (DmTrxR) which has a shorter N-terminal extension with low identity (30%), and accordingly lacks the cysteines in this protein portion that TrxRs from other species in this clade possess (Supplementary. Fig. S11). Interestingly, *D. medinensis* is the only worm in clade IIIc that has a free-living/free-swimming larval stage with an aquatic intermediate host (copepods) as part of its life cycle. All the other parasitic nematodes belonging to clade IIIc utilize blood-feeding insects or mites as their intermediate hosts/vectors. Like other biochemical pathways that evolved differences when filaria diverged from *D. medinensis*, the filarial TrxRs in clade IIIc may have also undergone changes that conferred a biochemical advantage with the fusion of the Grx-TrxR domain [34], and it is possible that the corresponding redox wiring is somehow linked to the nematode parasitic behavior in their intermediate hosts.

Three TrxR isoforms exist in mammals and isoform III has been identified as a Grx-TrxR fusion protein [77]. This latter isoform has a specific distribution in mammalian tissues and likely a specific function, being highly expressed in testis and involved in sperm maturation [78,79]. Moreover, a splice variant of TrxR isoform I (TrxR v3), bearing a Grx-like domain, has been found in humans and highly expressed in some cancer lines and, again, in testis [80]. In this case, the Grx domain has an atypical active site (CTRC) and lacks Grx activity; it is found that TrxR v3 has a strong affinity for membrane rafts and the capacity to induce actin and tubulin polymerization, thus promoting a prominent formation of cell membrane protrusions, suggesting a role in cancer progression in mammals [81,82]. Given the diverse and poorly characterized roles of TrxR isoenzymes containing a Grx domain, deeper -omic analyses of parasite-derived TrxRs are needed to better understand the role of this additional domain in the parasite’s life cycle and host interactions.

## 3 Conclusions and perspectives

More effective therapeutic options for the infections caused by filarial nematodes are needed. Current therapies are almost solely targeted towards larval stages. There is an acute need for new pharmacological substances effective against adult worms, which confer persistence of the infection and contribute to permanent disability. In efforts to find new and selective pharmacological treatments we present in-depth studies to further define the Trx system in *B. malayi* and *O. volvulus*. The functional properties of respective TrxRs and the inhibitory effects of previously identified TrxR inhibitors have been investigated. Auranofin has been established as a hTrxR inhibitor, so it was of interest to understand the differences in its effects on mammalian and filarial TrxR variants. More recently, TRi-1 and TRi-2, compounds that have varying selectivity for hTrxR isoforms, have been characterized, and their effects on the filarial TrxRs are reported here for the first time. Further investigations into the binding mechanism and specificity of these and other similar compounds could provide important insights into the host-parasite relationships driven by redox signalling and could expand the therapeutic tools available for filarial diseases.

Another key finding of the present work is that it is now clear that Grx-TrxR fusions are a recurrent theme in parasitic worms, for example cestodes (e.g., *Echinococcus* and *Taenia* spp.), trematodes (e.g., *Fasciola* and *Schistosoma* spp.) and now also in filarial nematodes of clade IIIc. The major difference between TrxR proteins is that the Grx-TrxR fusion proteins in parasitic platyhelminths are endowed with TrxR, GR, and Grx activities reflecting the fact that TGR is the sole enzyme that supports both Trx and GSH pathways in these organisms. In contrast, in clade IIIc nematodes, the Grx-TrxR fusion proteins do not have GR or Grx activity, in line with existence of two distinct NADPH-dependent flavoreductases supporting the GSH and Trx pathways in this phylum and the presence of canonical Grxs in the genomes of these parasites. Thus, we hypothesize that the Grx-TrxR fusion proteins in clade IIIc nematodes have a unique functional role. This is supported by our findings that the Grx domain is in redox communication with the TrxR domain, as occurs in SmTGR, shown by the adopted conformation of the C-terminus observed in the crystal structure induced by the adventitious arrangement of the subunits in the crystal lattice.

## 4 Materials and methods

### 4.1 Materials

Isopropyl β-d-1-thiogalactopyranoside (IPTG), sodium selenite (Na_2_SeO_3_), l-cysteine, ampicillin, chloramphenicol, riboflavin, lysozyme, Tris(hydroxymethyl) aminomethane (Tris), 1,4-Dithiothreitol (DTT), β-mercaptoethanol (βme), potassium phosphate, sodium chloride (NaCl), imidazole, flavin adenine dinucleotide (FAD), Auranofin were from Sigma-Aldrich. 2-methyl-2,4-pentandiol v/v 100% (MPD) from Molecular Dimension. EDTA was from Euroclone. Phenylmethanesulfonylfluoride (PMSF) was from ICN Biomedicals Inc. NADPH, Tetrasodium salt was from Roche and/or Sigma. 5,5′-dithiobis-(2-nitrobenzoic acid) (DTNB) and bovine pancreas insulin were obtained from Sigma. TRi-1 and TRi-2 were obtained from Oblique Therapeutics.

### 4.2 Expression and purification of recombinant cytoplasmatic BmTrxR and OvTrxR isoforms

The BmTrxR (isoform B) gene was cloned into pET100 expression vector, expressed and purified as reported previously [28]. Expression was performed BL21(DE3) cells co-transformed with pSUABC plasmid, necessary for correct selenocysteine-containing protein expression [61].

The BmTrxR (isoform D) and OvTrxR were produced in Sec enriched form for crystallography and kinetic studies. The genes were cloned into pABC2a vector, which yielded both the proteins fused with N-terminal His- and SUMO-tag. The plasmid (pABC2a-BmTrxR) was subsequently transformed into *E*. *coli* strain C321.ΔA [83] Protein expression, purification, and tag removal were as described [49,84].

The BmTrxR (isoform D) C22S mutant was generated using the Q5® Site-Directed Mutagenesis Kit from New England Biolabs with the mutagenic primers F-AAGAGCGCGTcCGAGGAACGTATC and R-GAAAACCGCGTCCGCCAG in pABC2a-BmTrxR. Protein expression, purification, and tag removal were performed the same as the wild-type enzyme.

### 4.3 Enzyme assay methods

Enzyme variants and Trxs recombinantly produced as described above for high Sec contents were used for all experiments, where BmTrxR and OvTrxR refers to the full-length isoforms D. Enzyme activity measurements were performed using a spectrophotometer. The enzymatic concentrations used for these assays were verified using wavelength scanning (ε_FAD_ = 11,300 M^−1^cm^−1^) since each subunit of the enzyme contains a FAD cofactor which has a maximum absorbance at 463 nm. NADPH consumption monitoring used ΔA_340_ = 6,220 M^−1^cm^−1^ and TNB formation monitoring used ΔA_412_ = 13,600 M^−1^cm^−1^. Experiments performed in microplate format used standard curves of NADPH consumption and TNB formation instead. All reactions were carried out in TES buffer, pH 7.5, containing 50 mM Tris-HCl, pH 7.5, 2 mM EDTA and 0.5 mM NaCl. The collected raw data was analyzed and used for plotting the figures of enzyme activity by using Prism GraphPad Software.

### 4.4 Enzyme kinetic assays with DTNB, GSSG, thioredoxin isoforms

Kinetic parameters of human and parasite TrxR enzymes were determined as previously described [85]. The speed of DTNB reduction by BmTrxR and OvTrxR was tested by adding 10 nM OvTrxR or 20 nM BmTrxR, 250 μM NADPH and 10 μM–200 μM DTNB, and following TNB formation at A_412_. Experiments were performed in cuvettes (n = 3) and enzyme velocity calculated based on ε_412_ = 13,600 M^−1^cm^−1^. Reduction of GSSG was measured in a reaction containing 15 nM BmTrxR, OvTrxR or hTrxR1, or 5 nM GR, with or without 25 μM BmTrx, OvTrx1 or hTrx1, in the presence of 1 mM GSSG and 250 μM NADPH, by following NADPH consumption at A_340_ (n = 2). The speed of reduction of filarial and human thioredoxin variants by BmTrxR and OvTrxR was assessed by measuring NADPH consumption linked to insulin reduction using 20 nM, 40 nM or 80 nM OvTrxR, BmTrxR, and hTrxR1, 160 μM insulin, 250 μM NADPH and 1.25 μM–25 μM BmTrx, OvTrx1, hTrx1, and following A_340_. Experiments were performed in duplicates in microplates and enzyme velocity calculated based on a standard curve for NADPH. Grx activity was determined as described [66]), with 1 mM GSH, 0.4 mM NADPH, and 6 μg/ml glutathione reductase in 2 mM EDTA, 0.1 mg/ml BSA and 0.1 M Tris-Cl, pH 8.0. The disulfide substrate HED was added to a final concentration of 0.7 mM. When BmTrxR (40 nM) was added the change in A_340_ was monitored.

BmTrxR C22S enzymatic assays were carried out with 4 nM enzyme, using both 10–500 μM NADPH at saturating concentration of DTNB and 30–2000 μM DTNB or 10–50 μM of SmTrx at saturating concentration of NADPH, on a Thermo Multiskan Spectrum plate reader. Assays were done in triplicate. Apparent Michaelis-Menten parameters were calculated based on the nonlinear fit of the velocity vs substrate concentration curves to the Michaelis-Menten equation, carried out in GraphPad Prism.

### 4.5 Inhibition of BmTrxR and OvTrxR

To assess inhibition by auranofin, TRi-1 and TRi-2, 25 nM BmTrxR or OvTrxR was allowed to react with 1 nM-1 μM of auranofin, TRi-1 or TRi-2, or 1% v/v DMSO, 250 μM NADPH and 0.1 mg/ml BSA for 30 min. Upon addition of 1 mM DTNB, TNB formation at A_412_ was followed. Experiments were carried out in duplicate. IC_50_ values were determined based on the fit of the normalized enzyme activity vs inhibitor concentration curve to a three-parameter nonlinear dose-response equation, carried out in GraphPad Prism.

### 4.6 Crystallization of BmTrxR isoforms B and D

Both BmTrxR variants used in this study were dialyzed and concentrated (Amicon Ultra-30 k) in 20 mM Hepes or MOPS, pH = 7, 200 mM NaCl, 1 mM EDTA, 0.02% Tween 20 and concentrated to setup crystallization trials. Crystals of the apo form of the protein grew in hanging vapor-diffusion method at 21 °C after 7 days in a drop formed by 1 μl of protein (5–15 mg/ml) and 1 μl of well solution containing 14–22% (v/v) MPD, 0.1 M Tris/HCl pH = 7.5–8.2, 5 mM of reducing agents (DTT or β-ME). Addition to well solutions of 5% (v/v) of PEG 200 or 400 improved crystal quality and X-ray diffraction. To obtain BmTrxR in complex with NADPH, apo-crystals were soaked with 1 mM NADPH for 72 h. The BmTrxR-Auranofin crystals were obtained washing twice in well solutions without reducing agents and then soaked with 300 μM of Auranofin, dissolved in DMSO, and 500 μM NADPH for 72 h. All crystals were cryoprotected with 25% (v/v) MPD and were flash frozen in liquid nitrogen.

### 4.7 Data collection, processing, model building and refinement

Diffraction data from crystal were collected at ELETTRA synchrotron (Trieste, Italy) at XRD2 beamline, at a wavelength of 1.0 Å and processed by XDS [86]. Data for BmTrxR-C22S mutant and BmTrxR isoform B (10–20% Sec content) were collected at ESRF (Grenoble, FR). Crystals belong to the C2221 space group with unit cell dimensions of a = 146.6 b = 260.1 c = 129.0 with three subunits in the asymmetric unit. The crystallographic structure was solved by molecular replacement using the program PHASER MR [87] of the CCP4 suite and the model was built starting from two search models: the structure of the human TrxR (57% identity with residue 103–598; PDB ID: 3QFA) and the crystal structure of glutaredoxin from *Clostridium oremlandii* (28% identity with residue 1–102 of BmTrxR; PDB ID: 4TR1). Model building and refinement were performed using COOT [88] and Refmac [89]. Waters have been automatically and manually added with COOT. The reported structures have been refined using 0.01 and 0.015 for the BmTrxR-NADPH structure as weighting terms, jelly-body refinement with sigma 0.1, with isotropic B-factors, using TLS restrains (one group for each subunit) and including non-crystallographic symmetry (medium restrains).

### 4.8 Structural analysis

Structural analysis has been carried out using COOT [88]. Areaimol of the CCP4suite has been used to calculate the buried area between the Grx and TrxR domains.

### 4.9 Bioinformatic analysis

Preliminary multiple sequence alignment for the TrxR isoforms used ClustalOmega, with the visualization of consensus sequence and conservation level performed within Jalview [90]. Secondary structure predictions using JPred [91] were also carried out from within Jalview.

Preliminary multiple sequence alignment for the Trx isoforms used MAFFT [92]. Homology modelling has been performed using both Robetta (https://robetta.bakerlab.org) and Modeller [93]. Sequence analysis has been carried out at NPS@ (https://npsa-prabi.ibcp.fr/cgi-bin/npsa_automat.pl?page=/NPSAHLP/npsahlp_npsageneral.html). The 3D model in which the C-terminus has been completely built (Supplementary Fig. S2), has been carried out with COOT [88] and geometrically optimized using the structural idealization subroutine of Refmac (CCP4 suite; [89].

## References

[1] World Health Organization (2021). Lymphatic filariasis. https://www.who.int/news-room/fact-sheets/detail/lymphatic-filariasis.

[2] WHO (2021).

[3] World Health Organization (2019). Onchocerciasis. https://www.who.int/news-room/fact-sheets/detail/onchocerciasis.

[4] Chakraborty S., Gurusamy M., Zawieja D.C., Muthuchamy M. (2013 Jul). Lymphatic filariasis: perspectives on lymphatic remodeling and contractile dysfunction in filarial disease pathogenesis. Microcirculation.

[5] Taylor M.J., Hoerauf A., Bockarie M. (2010 Oct 2). Lymphatic filariasis and onchocerciasis. Lancet.

[6] Boussinesq M., Gardon J., Gardon-Wendel N., Chippaux J.-P. (2003). Clinical picture, epidemiology and outcome of loa-associated serious adverse events related to mass ivermectin treatment of onchocerciasis in Cameroon. Filaria J..

[7] Geary T.G., Mackenzie C.D. (2011 Aug). Progress and challenges in the discovery of macrofilaricidal drugs. Expert Rev. Anti Infect. Ther..

[8] Hawryluk N.A. (2020 Apr 10). Macrofilaricides: an unmet medical need for filarial diseases. ACS Infect. Dis..

[9] Hoerauf A., Volkmann L., Hamelmann C., Adjei O., Autenrieth I.B., Fleischer B., Büttner D.W. (2000 Apr 8). Endosymbiotic bacteria in worms as targets for a novel chemotherapy in filariasis. Lancet.

[10] Taylor M.J., Makunde W.H., McGarry H.F., Turner J.D., Mand S., Hoerauf A. (2005 Jun 18-24). Macrofilaricidal activity after doxycycline treatment of Wuchereria bancrofti: a double-blind, randomised placebo-controlled trial. Lancet.

[11] Wan Sulaiman W.A., Kamtchum-Tatuene J., Mohamed M.H., Ramachandran V., Ching S.M., Sazlly Lim S.M., Hashim H.Z., Inche Mat L.N., Hoo F.K., Basri H. (2019 Jun). Anti-Wolbachia therapy for onchocerciasis & lymphatic filariasis: current perspectives. Indian J. Med. Res..

[12] Joardar N., Sinha Babu S.P. (2020 Jan 1). A review on the druggability of a thiol-based enzymatic antioxidant thioredoxin reductase for treating filariasis and other parasitic infections. Int. J. Biol. Macromol..

[13] Joardar N., Guevara-Flores A., Martínez-González J.J., Sinha Babu S.P. (2020 Dec 15). Thiol antioxidant thioredoxin reductase: a prospective biochemical crossroads between anticancer and antiparasitic treatments of the modern era. Int. J. Biol. Macromol..

[14] Kuntz A.N., Davioud-Charvet E., Sayed A.A., Califf L.L., Dessolin J., Arnér E.S., Williams D.L. (2007 Jun). Thioredoxin glutathione reductase from *Schistosoma mansoni*: an essential parasite enzyme and a key drug target. PLoS Med..

[15] Sayed A.A., Simeonov A., Thomas C.J., Inglese J., Austin C.P., Williams D.L. (2008 Apr). Identification of oxadiazoles as new drug leads for the control of schistosomiasis. Nat. Med..

[16] Williams D.L., Bonilla M., Gladyshev V.N., Salinas G. (2013 Sep 1). Thioredoxin glutathione reductase-dependent redox networks in platyhelminth parasites. Antioxidants Redox Signal..

[17] Angelucci F., Miele A.E., Ardini M., Boumis G., Saccoccia F., Bellelli A. (2016 Mar-Apr). Typical 2-Cys peroxiredoxins in human parasites: several physiological roles for a potential chemotherapy target. Mol. Biochem. Parasitol..

[18] Tripathi T., Suttiprapa S., Sripa B. (2017 Aug). Unusual thiol-based redox metabolism of parasitic flukes. Parasitol. Int..

[19] Lyu H., Petukhov P.A., Banta P.R., Jadhav A., Lea W.A., Cheng Q., Arnér E.S.J., Simeonov A., Thatcher G.R.J., Angelucci F., Williams D.L. (2020 Mar 13). Characterization of lead compounds targeting the selenoprotein thioredoxin glutathione reductase for treatment of schistosomiasis. ACS Infect. Dis..

[20] Boumis G., Giardina G., Angelucci F., Bellelli A., Brunori M., Dimastrogiovanni D., Saccoccia F., Miele A.E. (2012 Sep 7). Crystal structure of Plasmodium falciparum thioredoxin reductase, a validated drug target. Biochem. Biophys. Res. Commun..

[21] Fernandes A.P., Holmgren A. (2004 Feb). Glutaredoxins: glutathione-dependent redox enzymes with functions far beyond a simple thioredoxin backup system. Antioxidants Redox Signal..

[22] Deponte M. (2013 May). Glutathione catalysis and the reaction mechanisms of glutathione-dependent enzymes. Biochim. Biophys. Acta.

[23] Miller C.G., Holmgren A., Arnér E.S.J., Schmidt E.E. (2018 Nov 1). NADPH-dependent and -independent disulfide reductase systems. Free Radic. Biol. Med..

[24] Lu J., Vlamis-Gardikas A., Kandasamy K., Zhao R., Gustafsson T.N., Engstrand L., Hoffner S., Engman L., Holmgren A. (2013 Apr). Inhibition of bacterial thioredoxin reductase: an antibiotic mechanism targeting bacteria lacking glutathione. Faseb. J..

[25] Andrade R.M., Reed S.L. (2015 Sep 30). New drug target in protozoan parasites: the role of thioredoxin reductase. Front. Microbiol..

[26] Müller S., Gilberger T.W., Fairlamb A.H., Walter R.D. (1997). Molecular characterization and expression of Onchocerca volvulus glutathione reductase. Biochem. J..

[27] Fritz-Wolf K., Kehr S., Stumpf M., Rahlfs S., Becker K. (2011 Jul 12). Crystal structure of the human thioredoxin reductase-thioredoxin complex. Nat. Commun..

[28] Bulman C.A., Bidlow C.M., Lustigman S., Cho-Ngwa F., Williams D., Rascón A.A., Tricoche N., Samje M., Bell A., Suzuki B., Lim K.C., Supakorndej N., Supakorndej P., Wolfe A.R., Knudsen G.M., Chen S., Wilson C., Ang K.H., Arkin M., Gut J., Franklin C., Marcellino C., McKerrow J.H., Debnath A., Sakanari J.A. (2015 Feb 20). Repurposing auranofin as a lead candidate for treatment of lymphatic filariasis and onchocerciasis. PLoS Neglected Trop. Dis..

[29] Feng L., Pomel S., Latre de Late P., Taravaud A., Loiseau P.M., Maes L., Cho-Ngwa F., Bulman C.A., Fischer C., Sakanari J.A., Ziniel P.D., Williams D.L., Davioud-Charvet E. (2020 Nov 1). Repurposing auranofin and evaluation of a new gold(I) compound for the search of treatment of human and cattle parasitic diseases: from Protozoa to helminth infections. Molecules.

[30] Alger H.M., Williams D.L. (2002 Apr 30). The disulfide redox system of Schistosoma mansoni and the importance of a multifunctional enzyme, thioredoxin glutathione reductase. Mol. Biochem. Parasitol..

[31] Angelucci F., Dimastrogiovanni D., Boumis G., Brunori M., Miele A.E., Saccoccia F., Bellelli A. (2010 Oct 15). Mapping the catalytic cycle of *Schistosoma mansoni* thioredoxin glutathione reductase by X-ray crystallography. J. Biol. Chem..

[32] Huang H.H., Day L., Cass C.L., Ballou D.P., Williams C.H., Williams D.L. (2011 Jul 5). Investigations of the catalytic mechanism of thioredoxin glutathione reductase from *Schistosoma mansoni*. Biochemistry.

[33] Otero L., Bonilla M., Protasio A.V., Fernández C., Gladyshev V.N., Salinas G. (2010 Apr 13). Thioredoxin and glutathione systems differ in parasitic and free-living platyhelminths. BMC Genom..

[34] International Helminth Genomes Consortium (2019 Jan). Comparative genomics of the major parasitic worms. Nat. Genet..

[35] Sandalova T., Zhong L., Lindqvist Y., Holmgren A., Schneider G. (2001 Aug 14). Three-dimensional structure of a mammalian thioredoxin reductase: implications for mechanism and evolution of a selenocysteine-dependent enzyme. Proc. Natl. Acad. Sci. U. S. A..

[36] Kunchithapautham K., Padmavathi B., Narayanan R.B., Kaliraj P., Scott A.L. (2003 Jul). Thioredoxin from *Brugia malayi*: defining a 16-kilodalton class of thioredoxins from nematodes. Infect. Immun..

[37] Zhang X., Lu J., Ren X., Du Y., Zheng Y., Ioannou P.V., Holmgren A. (2015 Dec). Oxidation of structural cysteine residues in thioredoxin 1 by aromatic arsenicals enhances cancer cell cytotoxicity caused by the inhibition of thioredoxin reductase 1. Free Radic. Biol. Med..

[38] Sun Q.A., Su D., Novoselov S.V., Carlson B.A., Hatfield D.L., Gladyshev V.N. (2005 Nov 8). Reaction mechanism and regulation of mammalian thioredoxin/glutathione reductase. Biochemistry.

[39] Gromer S., Merkle H., Schirmer R.H., Becker K. (2002). Human placenta thioredoxin reductase: preparation and inhibitor studies. Methods Enzymol..

[40] Arnér E.S.J. (2018). Selective evaluation of thioredoxin reductase enzymatic activities. Methods Mol. Biol..

[41] Boumis G., Angelucci F., Bellelli A., Brunori M., Dimastrogiovanni D., Miele A.E. (2011 Jun). Structural and functional characterization of Schistosoma mansoni Thioredoxin. Protein Sci..

[42] Xu J., Cheng Q., Arnér E.S. (2016 May). Details in the catalytic mechanism of mammalian thioredoxin reductase 1 revealed using point mutations and juglone-coupled enzyme activities. Free Radic. Biol. Med..

[43] Gromer S., Arscott L.D., Williams C.H., Schirmer R.H., Becker K. (1998). Human placenta thioredoxin reductase. Isolation of the selenoenzyme, steady state kinetics, and inhibition by therapeutic gold compounds. J. Biol. Chem..

[44] Rigobello M.P., Folda A., Baldoin M.C., Scutari G., Bindoli A. (2005 Jul). Effect of auranofin on the mitochondrial generation of hydrogen peroxide. Role of thioredoxin reductase. Free Radic. Res..

[45] Stafford W.C., Peng X., Olofsson M.H., Zhang X., Luci D.K., Lu L., Cheng Q., Trésaugues L., Dexheimer T.S., Coussens N.P., Augsten M., Ahlzén H.M., Orwar O., Östman A., Stone-Elander S., Maloney D.J., Jadhav A., Simeonov A., Linder S., Arnér E.S.J. (2018 Feb 14). Irreversible inhibition of cytosolic thioredoxin reductase 1 as a mechanistic basis for anticancer therapy. Sci. Transl. Med..

[46] Zhang X., Selvaraju K., Saei A.A., D'Arcy P., Zubarev R.A., Arnér E.S., Linder S. (2019 Jul). Repurposing of auranofin: thioredoxin reductase remains a primary target of the drug. Biochimie.

[47] Angelucci F., Sayed A.A., Williams D.L., Boumis G., Brunori M., Dimastrogiovanni D., Miele A.E., Pauly F., Bellelli A. (2009 Oct 16). Inhibition of *Schistosoma mansoni* thioredoxin-glutathione reductase by auranofin: structural and kinetic aspects. J. Biol. Chem..

[48] Sabatier P., Beusch C.M., Gencheva R., Cheng Q., Zubarev R., Arnér E.S.J. (2021). Comprehensive chemical proteomics analyses reveal that the new TRi-1 and TRi-2 compounds are more specific thioredoxin reductase 1 inhibitors than auranofin. Redox Biol..

[49] Cheng Q., Arnér E.S. (2017 Mar 31). Selenocysteine insertion at a predefined UAG codon in a release factor 1 (RF1)-depleted *Escherichia coli* host strain bypasses species barriers in recombinant selenoprotein translation. J. Biol. Chem..

[50] Lee E.H., Kim H.Y., Hwang K.Y. (2014 Dec 15). The GSH- and GSSG-bound structures of glutaredoxin from. Clostridium oremlandii. Arch Biochem Biophys.

[51] Angelucci F., Miele A.E., Boumis G., Dimastrogiovanni D., Brunori M., Bellelli A. (2008 Aug 15). Glutathione reductase and thioredoxin reductase at the crossroad: the structure of *Schistosoma mansoni* thioredoxin glutathione reductase. Proteins.

[52] Silvestri I., Lyu H., Fata F., Boumis G., Miele A.E., Ardini M., Ippoliti R., Bellelli A., Jadhav A., Lea W.A., Simeonov A., Cheng Q., Arnér E.S.J., Thatcher G.R.J., Petukhov P.A., Williams D.L., Angelucci F. (2018 Aug 17). Fragment-based discovery of a regulatory site in thioredoxin glutathione reductase acting as "doorstop" for NADPH entry. ACS Chem. Biol..

[53] Fata F., Silvestri I., Ardini M., Ippoliti R., Di Leandro L., Demitri N., Polentarutti M., Di Matteo A., Lyu H., Thatcher G.R.J., Petukhov P.A., Williams D.L., Angelucci F. (2021 Jul 9). Probing the surface of a parasite drug target thioredoxin glutathione reductase using small molecule fragments. ACS Infect. Dis..

[54] Silvestri I., Lyu H., Fata F., Banta P.R., Mattei B., Ippoliti R., Bellelli A., Pitari G., Ardini M., Petukhova V., Thatcher G.R.J., Petukhov P.A., Williams D.L., Angelucci F. (2020 Feb 1). Ectopic suicide inhibition of thioredoxin glutathione reductase. Free Radic. Biol. Med..

[55] Cheng Q., Sandalova T., Lindqvist Y., Arnér E.S. (2009 Feb 6). Crystal structure and catalysis of the selenoprotein thioredoxin reductase 1. J. Biol. Chem..

[56] Ho B.K., Brasseur R. (2005 Aug 16). The Ramachandran plots of glycine and pre-proline. BMC Struct. Biol..

[57] Collet J.F., Messens J. (2010 Oct). Structure, function, and mechanism of thioredoxin proteins. Antioxidants Redox Signal..

[58] Parker A.R., Petluru P.N., Nienaber V.L., Badger J., Leverett B.D., Jair K., Sridhar V., Logan C., Ayala P.Y., Kochat H., Hausheer F.H. (2015 Mar 18). Cysteine specific targeting of the functionally distinct peroxiredoxin and glutaredoxin proteins by the investigational disulfide BNP7787. Molecules.

[59] Lillig C.H., Berndt C. (2013 May 1). Glutaredoxins in thiol/disulfide exchange. Antioxidants Redox Signal..

[60] Roos G., Foloppe N., Messens J. (2013 Jan 1). Understanding the pK(a) of redox cysteines: the key role of hydrogen bonding. Antioxidants Redox Signal..

[61] Arnér E.S., Sarioglu H., Lottspeich F., Holmgren A., Böck A. (1999 Oct 8). High-level expression in *Escherichia coli* of selenocysteine-containing rat thioredoxin reductase utilizing gene fusions with engineered bacterial-type SECIS elements and co-expression with the selA, selB and selC genes. J. Mol. Biol..

[62] Marino S.M., Gladyshev V.N. (2010 Dec 17). Cysteine function governs its conservation and degeneration and restricts its utilization on protein surfaces. J. Mol. Biol..

[63] Marino S.M., Gladyshev V.N. (2012 Feb 10). Analysis and functional prediction of reactive cysteine residues. J. Biol. Chem..

[64] Bonilla M., Denicola A., Novoselov S.V., Turanov A.A., Protasio A., Izmendi D., Gladyshev V.N., Salinas G. (2008 Jun 27). Platyhelminth mitochondrial and cytosolic redox homeostasis is controlled by a single thioredoxin glutathione reductase and dependent on selenium and glutathione. J. Biol. Chem..

[65] Rendón J.L., del Arenal I.P., Guevara-Flores A., Uribe A., Plancarte A., Mendoza-Hernández G. (2004 Jan). Purification, characterization and kinetic properties of the multifunctional thioredoxin-glutathione reductase from *Taenia crassiceps* metacestode (cysticerci). Mol. Biochem. Parasitol..

[66] Holmgren A., Aslund F. (1995). Glutaredoxin. Methods Enzymol..

[67] Begas P., Staudacher V., Deponte M. (2015). Systematic re-evaluation of the bis(2-hydroxyethyl)disulfide (HEDS) assay reveals an alternative mechanism and activity of glutaredoxins. Chem. Sci..

[68] Zimmermann J., Oestreicher J., Hess S., Herrmann J.M., Deponte M., Morgan B. (2020). One cysteine is enough: a monothiol Grx can functionally replace all cytosolic Trx and dithiol Grx. Redox Biol..

[69] Fernandes A.P., Fladvad M., Berndt C., Andrésen C., Lillig C.H., Neubauer P., Sunnerhagen M., Holmgren A., Vlamis-Gardikas A. (2005 Jul 1). A novel monothiol glutaredoxin (Grx4) from Escherichia coli can serve as a substrate for thioredoxin reductase. J. Biol. Chem..

[70] Liedgens L., Zimmermann J., Wäschenbach L., Geissel F., Laporte H., Gohlke H., Morgan B., Deponte M. (2020 Apr 7). Quantitative assessment of the determinant structural differences between redox-active and inactive glutaredoxins. Nat. Commun..

[71] Angiulli G., Lantella A., Forte E., Angelucci F., Colotti G., Ilari A., Malatesta F. (2015 Sep). *Leishmania infantum* trypanothione reductase is a promiscuous enzyme carrying an NADPH:O2 oxidoreductase activity shared by glutathione reductase. Biochim. Biophys. Acta.

[72] Argyrou A., Blanchard J.S. (2004). Flavoprotein disulfide reductases: advances in chemistry and function. Prog. Nucleic Acid Res. Mol. Biol..

[73] Fritz-Wolf K., Urig S., Becker K. (2007 Jun 29). The structure of human thioredoxin reductase 1 provides insights into C-terminal rearrangements during catalysis. J. Mol. Biol..

[74] Pickering I.J., Cheng Q., Rengifo E.M., Nehzati S., Dolgova N.V., Kroll T., Sokaras D., George G.N., Arnér E.S.J. (2020 Mar 2). Direct observation of methylmercury and auranofin binding to selenocysteine in thioredoxin reductase. Inorg. Chem..

[75] Forneris F., Orru R., Bonivento D., Chiarelli L.R., Mattevi A. (2009 May). ThermoFAD, a Thermofluor-adapted flavin ad hoc detection system for protein folding and ligand binding. FEBS J..

[76] Pasquo A., Consalvi V., Knapp S., Alfano I., Ardini M., Stefanini S., Chiaraluce R. (2012). Structural stability of human protein tyrosine phosphatase ρ catalytic domain: effect of point mutations. PLoS One.

[77] Arnér E.S. (2009 Jun). Focus on mammalian thioredoxin reductases--important selenoproteins with versatile functions. Biochim. Biophys. Acta.

[78] Su D., Novoselov S.V., Sun Q.A., Moustafa M.E., Zhou Y., Oko R., Hatfield D.L., Gladyshev V.N. (2005 Jul 15). Mammalian selenoprotein thioredoxin-glutathione reductase. Roles in disulfide bond formation and sperm maturation. J. Biol. Chem..

[79] Brandstaedter C., Fritz-Wolf K., Weder S., Fischer M., Hecker B., Rahlfs S., Becker K. (2018 Feb). Kinetic characterization of wild-type and mutant human thioredoxin glutathione reductase defines its reaction and regulatory mechanisms. FEBS J..

[80] Damdimopoulou P.E., Miranda-Vizuete A., Arnér E.S., Gustafsson J.A., Damdimopoulos A.E. (2009 Oct). The human thioredoxin reductase-1 splice variant TXNRD1_v3 is an atypical inducer of cytoplasmic filaments and cell membrane filopodia. Biochim. Biophys. Acta.

[81] Dammeyer P., Damdimopoulos A.E., Nordman T., Jiménez A., Miranda-Vizuete A., Arnér E.S. (2008 Feb 1). Induction of cell membrane protrusions by the N-terminal glutaredoxin domain of a rare splice variant of human thioredoxin reductase 1. J. Biol. Chem..

[82] Cebula M., Moolla N., Capovilla A., Arnér E.S.J. (2013 Apr 5). The rare TXNRD1_v3 ("v3") splice variant of human thioredoxin reductase 1 protein is targeted to membrane rafts by N-acylation and induces filopodia independently of its redox active site integrity. J. Biol. Chem..

[83] Wannier T.M., Kunjapur A.M., Rice D.P., McDonald M.J., Desai M.M., Church G.M. (2018 Mar 20). Adaptive evolution of genomically recoded Escherichia coli. Proc. Natl. Acad. Sci. U. S. A..

[84] Cheng Q., Roveri A., Cozza G., Bordin L., Rohn I., Schwerdtle T., Kipp A., Ursini F., Maiorino M., Miotto G., Arnér E.S.J. (2021 Oct). Production and purification of homogenous recombinant human selenoproteins reveals a unique codon skipping event in E. coli and GPX4-specific affinity to bromosulfophthalein. Redox Biol..

[85] Arnér E.S., Holmgren A. (2001 May). Measurement of thioredoxin and thioredoxin reductase. Curr. Protoc. Toxicol..

[86] Kabsch W.X.D.S. (2010). Acta Crystallogr. D. Biol. Crystallogr..

[87] McCoy A.J., Grosse-Kunstleve R.W., Adams P.D., Winn M.D., Storoni L.C., Read R.J. (2007 Aug 1). Phaser crystallographic software. J. Appl. Crystallogr..

[88] Emsley P., Lohkamp B., Scott W.G., Cowtan K. (2010 Apr). Features and development of coot. Acta Crystallogr. D. Biol. Crystallogr..

[89] Nicholls R.A., Long F., Murshudov G.N. (2012 Apr). Low-resolution refinement tools in REFMAC5. Acta Crystallogr. D. Biol. Crystallogr..

[90] Waterhouse A.M., Procter J.B., Martin D.M., Clamp M., Barton G.J. (2009 May 1). Jalview Version 2--a multiple sequence alignment editor and analysis workbench. Bioinformatics.

[91] Drozdetskiy A., Cole C., Procter J., Barton G.J. (2015 Jul 1). JPred4: a protein secondary structure prediction server. Nucleic Acids Res..

[92] Katoh K., Misawa K., Kuma K., Miyata T. (2002 Jul 15). MAFFT: a novel method for rapid multiple sequence alignment based on fast Fourier transform. Nucleic Acids Res..

[93] Webb B., Sali A. (2016 Jun 20). Comparative protein structure modeling using MODELLER. Curr. Protoc. Bioinformatics.

