# Supplementary material for: Biochemical and structural characterizations of thioredoxin reductase selenoproteins of the parasitic filarial nematodes Brugia malayi and Onchocerca volvulus
Source: Redox Biol. 2022 Mar 4;51:102278. doi: 10.1016/j.redox.2022.102278 (PMC8914392; doi:10.1016/j.redox.2022.102278)
Supplement: Multimedia component 1 [file mmc1.docx]

**SUPPLEMENTARY DATA AND FIGURES**

**Biochemical and Structural Characterizations of Thioredoxin Reductase Selenoproteins of the Parasitic Filarial Nematodes *Brugia malayi* and *Onchocerca volvulus***

Francesca Fata^#,1^, Radosveta Gencheva^#,2^, Qing Cheng^2^, Rachel Lullo^3^, Matteo Ardini^1^, Ilaria Silvestri^1^, Federica Gabriele^1^, Rodolfo Ippoliti^1^, Christina A. Bulman^4^, Judy A. Sakanari^4^, David L. Williams^3,§^, Elias S.J. Arnér^§,2,5^, Francesco Angelucci^1,§,&^

^#^ Co-first authors

^§^ Co-senior authors

^&^ Corresponding author:

francesco.angelucci@univaq.it

^1^ Dept. of Life, Health and Environmental Sciences, University of L'Aquila, L’Aquila 67100, Italy

^2^ Division of Biochemistry, Department of Medical Biochemistry and Biophysics, Karolinska Institutet, Stockholm 17177, Sweden

^3^ Dept. of Microbial Pathogens and Immunity, Rush University Medical Center, Chicago, IL USA

^4^ Dept. of Pharmaceutical Chemistry, University of California, San Francisco, CA USA

^5^ Department of Selenoprotein Research, National Institute of Oncology, 1122 Budapest, Hungary

­

C

B

BmTrxR isoform

ATG

ATG

C

ATG

D

C

B

D

ChrX_scaffold_001

**Supplementary Figure S1.** **BmTrxR gene model and splicing variants.** Boxes represent exons, black lines intron regions. The fine lines represent splicing events. The 5’ ends of spliced isoforms are shown on the right. Sequences downstream are identical in these three variants. Splice variant D model was generated using Bm2025d.1 from WormBase ParaSite Version: WBPS16 (WS279).

BmTrxR_C MWHSRILMQCCTSWYAVRVGINIGVLEKSIGARRIKKHSLIIFTSKMYSEQSLTGFNRSK 60

BmTrxR_A ------------------------------------------------------------ 0

BmTrxR_B ------------------------------------------------------------ 0

BmTrxR_D ------------------------------------------------------------ 0

BmTrxR_C LEAFIRCYNWITCSRNVGRS--DVSRQPNSAVHNSCNYKSTSDKRSRRGNRSGREASMSP 118

BmTrxR_D ------------------------------------------------------------ 0

BmTrxR_B -------------MISVGLLFLGIFRNA----QAYLLRSNATDKRSRRGNRSGREASMSP 43

BmTrxR_D ---------------------------------------------------------MSP 3

BmTrxR_C IPNRVSSGSLADAVFKSACEERILLAYADYNPDMTKVVNLFSKYNETVNTVRVSNDAVKD 178

BmTrxR_A ------------------------------------------------------------ 0

BmTrxR_B IPNRVSSGSLADAVFKSACEERILLAYADYNPDMTKVVNLFSKYNETVNTVRVSNDAVKD 103

BmTrxR_D IPNRVSSGSLADAVFKSACEERILLAYADYNPDMTKVVNLFSKYNETVNTVRVSNDAVKD 63

BmTrxR_C ILEIVGWPSMPLIFVKGNCCGGFKELYQLEESGFLNEWLKEHEYDLAIVGGGSGGLAAAK 238

BmTrxR_A ------------------------------------------------------------ 0

BmTrxR_B ILEIVGWPSMPLIFVKGNCCGGFKELYQLEESGFLNEWLKEHEYDLAIVGGGSGGLAAAK 163

BmTrxR_D ILEIVGWPSMPLIFVKGNCCGGFKELYQLEESGFLNEWLKEHEYDLAIVGGGSGGLAAAK 123

BmTrxR_C EAVRLGKKVVCLDFVKPSAMGTTWGLGGTCVNVGCIPKKLMHQAALLGEYIEDAKKFGWE 298

BmTrxR_A ------------------------------------------------------------ 0

BmTrxR_B EAVRLGKKVVCLDFVKPSAMGTTWGLGGTCVNVGCIPKKLMHQAALLGEYIEDAKKFGWE 223

BmTrxR_D EAVRLGKKVVCLDFVKPSAMGTTWGLGGTCVNVGCIPKKLMHQAALLGEYIEDAKKFGWE 183

BmTrxR_C IPEGAIKLNWHQLKNAVQNHIASLNWGYRVQLKEKSVTYMNSYATFTGSHELSVKNKKGK 358

BmTrxR_A ---------------------------------------MNSYATFTGSHELSVKNKKGK 21

BmTrxR_B IPEGAIKLNWHQLKNAVQNHIASLNWGYRVQLKEKSVTYMNSYATFTGSHELSVKNKKGK 283

BmTrxR_D IPEGAIKLNWHQLKNAVQNHIASLNWGYRVQLKEKSVTYMNSYATFTGSHELSVKNKKGK 243

*********************

BmTrxR_C VEKVTADRFLIAVGLRPRFPDVPGALECCISSDDLFSLPYNPGKTLCVGASYVSLECAGF 418

BmTrxR_A VEKVTADRFLIAVGLRPRFPDVPGALECCISSDDLFSLPYNPGKTLCVGASYVSLECAGF 81

BmTrxR_B VEKVTADRFLIAVGLRPRFPDVPGALECCISSDDLFSLPYNPGKTLCVGASYVSLECAGF 343

BmTrxR_D VEKVTADRFLIAVGLRPRFPDVPGALECCISSDDLFSLPYNPGKTLCVGASYVSLECAGF 303

************************************************************

BmTrxR_C LKGIGNDVTVMVRSVLLRGFDQDMAERIKKHMTERGVKFVQCVPIKYERLKKPTDSEPGM 478

BmTrxR_A LKGIGNDVTVMVRSVLLRGFDQDMAERIKKHMTERGVKFVQCVPIKYERLKKPTDSEPGM 141

BmTrxR_B LKGIGNDVTVMVRSVLLRGFDQDMAERIKKHMTERGVKFVQCVPIKYERLKKPTDSEPGM 403

BmTrxR_D LKGIGNDVTVMVRSVLLRGFDQDMAERIKKHMTERGVKFVQCVPIKYERLKKPTDSEPGM 363

************************************************************

BmTrxR_C IRVHTMQEDEDGTKEVTEDFNTVLMAIGRDAMTDDLGLDVVGVNRAKSGKIIGRREQSVS 538

BmTrxR_A IRVHTMQEDEDGTKEVTEDFNTVLMAIGRDAMTDDLGLDVVGVNRAKSGKIIGRREQSVS 201

BmTrxR_B IRVHTMQEDEDGTKEVTEDFNTVLMAIGRDAMTDDLGLDVVGVNRAKSGKIIGRREQSVS 463

BmTrxR_D IRVHTMQEDEDGTKEVTEDFNTVLMAIGRDAMTDDLGLDVVGVNRAKSGKIIGRREQSVS 423

************************************************************

BmTrxR_C CPYVYAIGDVLYGSPELTPVAIQAGKVLMRRLFTGSSELTEYDKIPTTVFTPLEYGSCGL 598

BmTrxR_A CPYVYAIGDVLYGSPELTPVAIQAGKVLMRRLFTGSSELTEYDKIPTTVFTPLEYGSCGL 261

BmTrxR_B CPYVYAIGDVLYGSPELTPVAIQAGKVLMRRLFTGSSELTEYDKIPTTVFTPLEYGSCGL 523

BmTrxR_D CPYVYAIGDVLYGSPELTPVAIQAGKVLMRRLFTGSSELTEYDKIPTTVFTPLEYGSCGL 483

************************************************************

BmTrxR_C SEYSAIQKYGKENINVYHNVFIPLEYAVTERKEKTHCYCKLICLKNEQDLILGFHILTPN 658

BmTrxR_A SEYSAIQKYGKENINVYHNVFIPLEYAVTERKEKTHCYCKLICLKNEQDLILGFHILTPN 321

BmTrxR_B SEYSAIQKYGKENINVYHNVFIPLEYAVTERKEKTHCYCKLICLKNEQDLILGFHILTPN 583

BmTrxR_D SEYSAIQKYGKENINVYHNVFIPLEYAVTERKEKTHCYCKLICLKNEQDLILGFHILTPN 543

************************************************************

BmTrxR_C AGEITQGFAIALKFDAKKADFDRLIGIHPTVAENFTTLTLVKEDGQTLKATGCUG 713

BmTrxR_A AGEITQGFAIALKFDAKKADFDRLIGIHPTVAENFTTLTLVKEDGQTLKATGCUG 376

BmTrxR_B AGEITQGFAIALKFDAKKADFDRLIGIHPTVAENFTTLTLVKEDGQTLKATGCUG 638

BmTrxR_D AGEITQGFAIALKFDAKKADFDRLIGIHPTVAENFTTLTLVKEDGQTLKATGCUG 598

*******************************************************

**Supplementary Figure S2. BmTrxR isoforms A-D**. The canonical FAD active site motif is highlighted in yellow, while the N-terminal extensions of isoform B and C are in green and blue respectively. Isoform A is likely not expressed due to lacking the FAD active site motif. Isoform B is predicted to have a signal peptide at its N-terminus (probability 0.77) or, less probably, a mitochondrial transfer peptide (0.17); while isoform C has the N-terminal peptide predicted as “other” (0.77; indicating that the sequence likely does not have any kind of signal peptide) or, less probably, as mitochondrial transit peptide (0.1) or as signal peptide (0.13; <https://services.healthtech.dtu.dk/service.php?TargetP-2.0>).

***A***

***B***

BmTrx1 MADLLANINLKKADGTVKKGSDALANKKVVALYFSAHWCPPCRQFTPILKEFYEEVDDDQFEIVFVSLDHSEEDLNNYVKESHGNWYYVPFGSSEIEKLKNKYEVAGIPMLIVIKSDGNVITKNGRADVSGKAPPQTLSSWLAAA-

BmTrx2 MADLLANINLKKADGTVKKGSDALANKKVVALYFSAHWCPQCRQFTPILKEFYEEVDDDQFEIVFVSLDHSEEDLNNYVKESHGNWYYVPFGSSEIEKLKNKYEVAGIPMLIVIKSDGNVITKNGRADVSGKAPPQTLSSWLAAA-

OvTrx1 MADLLADVYLKKTDGAVKKGSDALANKTIVALYFSAHWCPPCRQFTPILKEFYEEVEDERFEIVFVSLDHSEKDLNNYLKESHGDWYHIPFGSDDIEKLKNKYEIAGIPMLIVIKSDGTVITKNGRADVSGKAPPQTLSKWLAAA-

OvTrx2 MADLLANVDLKKADGILKKGSDVLANKKVVALYFAAHWCPQCRRFTPSLKEFYKELNDDQFEIVFVSLDRSAEDLDNYLKEVHGDWYCIPFGSSEIEELKNKYEVAGIPMLIVIKSDGTVVTKNGRTDVSGKTPSEALSGWIAAAK

hTrx1 MVKQI------ESKTAFQEALDA-AGDKLVVVDFSATWCGPCKMIKP----FFHSLSEKYSNVIFLEVD-----------------------VDDCQDVASECEVKCMPTFQFFKKGQKV------GEFSG-ANKEKLEATINELV

*.. : ::. .::. *. *...:*.: *:* ** *: :.* *:..:.:. :::*:.:* .: :.: .: *: :* : .:*.. .* :.** : : *. :

**Supplementary Figure S3. Detailed alignments of the enzymes studied here.** In *A*, a detailed Clustal Omega alignment of the enzymes schematically shown in Fig. 1 is given, with conserved features, visualized in Jalview, and predicted by JPred secondary structure elements indicated. Grx active site, N-terminal redox active motifs and C-terminal redox active motifs marked in blue, orange and red respectively. UniProt entries are as follows: BmTrxR (UniProtKB - A0A0J9XPT5), OvTrxR (UniProtKB - A0A044TEU6), hTrxR (Accession: AAB35418.1) and SmTGR (Accession: AAK85233.1). In *B,* MAFFT alignments of BmTrx1 (Accession: AY117545), BmTrx2 (see main text), OvTrx1 (Accession: AY142708), OvTrx2 (Accession: AY142706) and hTrx1 (Accession: AAF87085.1) are shown, with Trx active sites marked in yellow and potentially structural cysteine residues marked in green.

Please, use the zoom feature in the digital version of this article to see all details in the alignment of panel A.


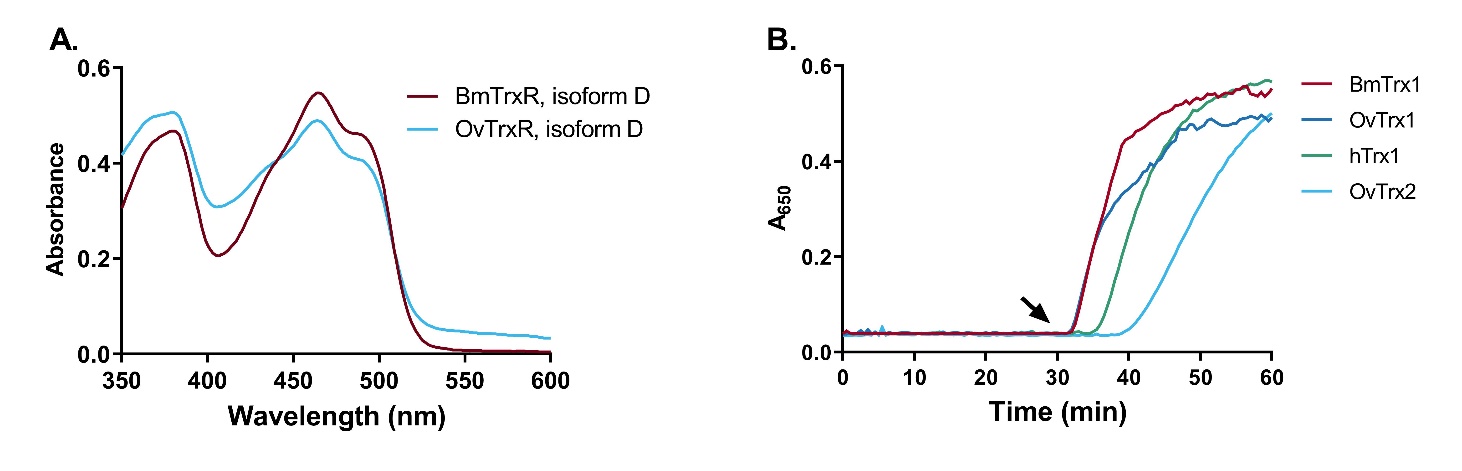


**Supplementary Figure S4**. Characterization of Trx and TrxR from *B. malayi* and *O. volvulus* by absorption spectra and insulin reduction capacity. (A) Absorption spectra of BmTrxR and OvTrxR. (B) Trx-mediated insulin reduction by DTT. Absorption at 650 nm was followed upon addition of 0.5 mM GSH to a reaction containing 5 µM Trx, 0.25 mM NADPH and 0.16 mM bovine insulin. After 30 min, 0.5 mM DTT (indicated by arrow) was added to the reaction and insulin reduction was continuously followed.


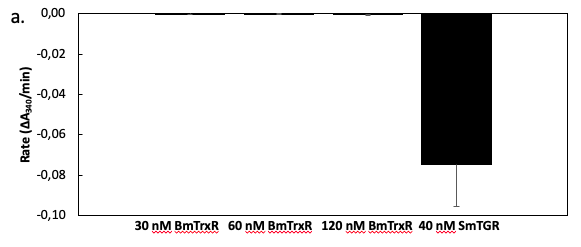


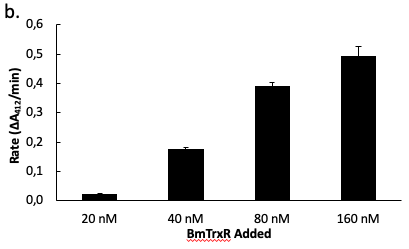


**Supplementary Fig. S5. Grx activity of BmTrxR.** (a) The Grx activity of recombinant BmTrxR was determined using the HEDS reduction assay (Holmgren A, Aslund F. Glutaredoxin. Methods Enzymol. 1995; 252:283-92. doi: 10.1016/0076-6879(95)52031-7. PMID: 7476363). (b) The TrxR activity of recombinant BmTrxR was determined using the DTNB assay as described in the text. The assay was done in triplicate with different amounts of BmTrxR added.


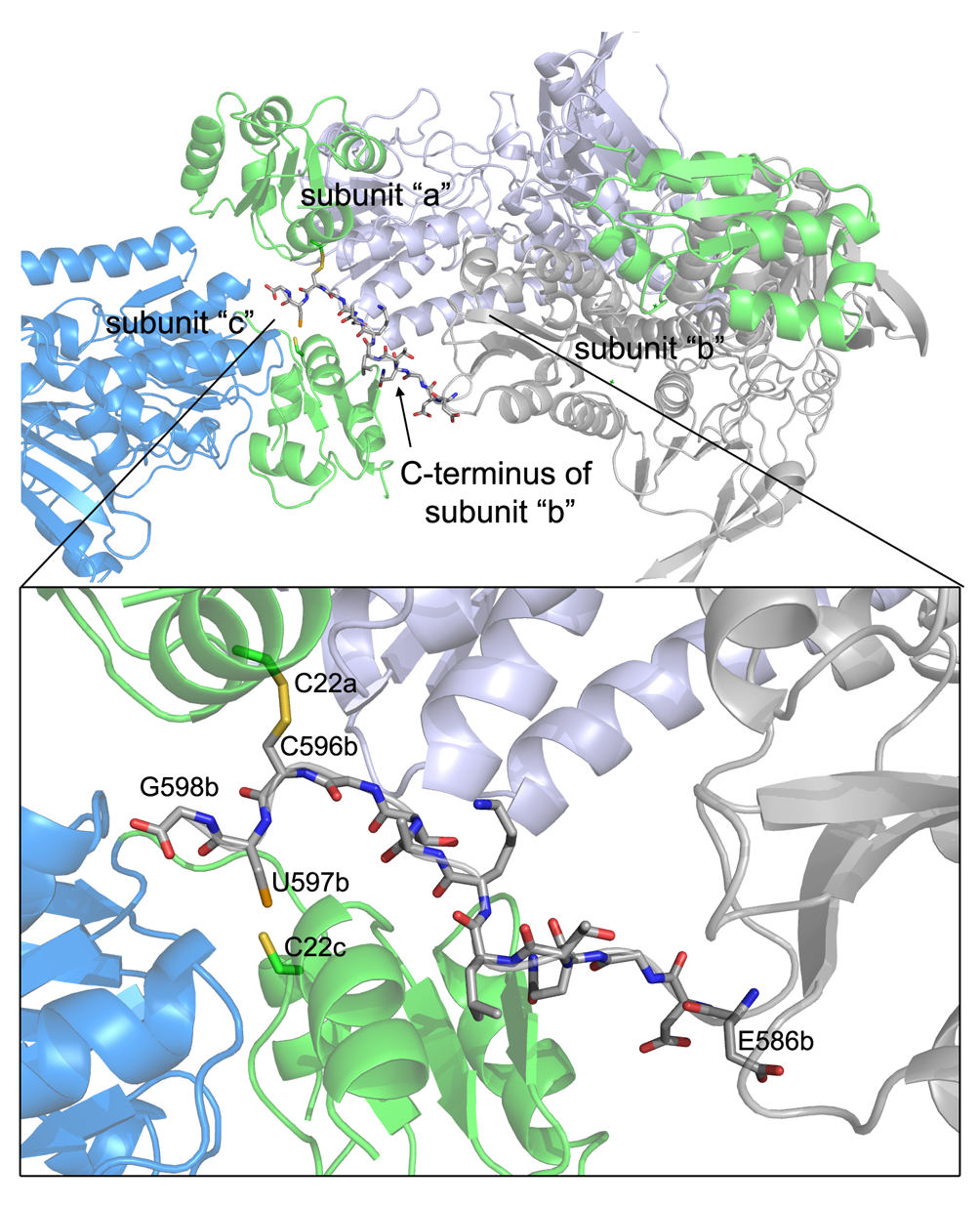


**Supplementary Figure S6**. A 3D model of BmTrxR in which the 8 missing residues from E587 to G595 were added and geometrically optimized to demonstrate that the electron density between C22a and C22c is the C-terminal residues of subunit “b”.

β_1_

MSPIPNRVSSGSLADAVFKSACEERILLAYADYNPDMTKVVNLFSKYNETVNTVRVSNDAVKDILEIVGW

α_5_

β_4_

β_2_

α_4_

α_3_

α_2_

α_1_

β_3_

PSMPLIFVKGNCCGGFKELYQLEESGFLNEWLKEHEYDLAIVGGGSGGLAAAKEAVRLGKKVVCLDFVKP

SAMGTTWGLGGTCVNVGCIPKKLMHQAALLGEYIEDAKKFGWEIPEGAIKLNWHQLKNAVQNHIASLNWG

YRVQLKEKSVTYMNSYATFTGSHELSVKNKKGKVEKVTADRFLIAVGLRPRFPDVPGALECCISSDDLFS

LPYNPGKTLCVGASYVSLECAGFLKGIGNDVTVMVRSVLLRGFDQDMAERIKKHMTERGVKFVQCVPIKY

ERLKKPTDSEPGMIRVHTMQEDEDGTKEVTEDFNTVLMAIGRDAMTDDLGLDVVGVNRAKSGKIIGRREQ

SVSCPYVYAIGDVLYGSPELTPVAIQAGKVLMRRLFTGSSELTEYDKIPTTVFTPLEYGSCGLSEYSAIQ

KYGKENINVYHNVFIPLEYAVTERKEKTHCYCKLICLKNEQDLILGFHILTPNAGEITQGFAIALKFDAK

KADFDRLIGIHPTVAENFTTLTLVKEDGQTLKATGCUG


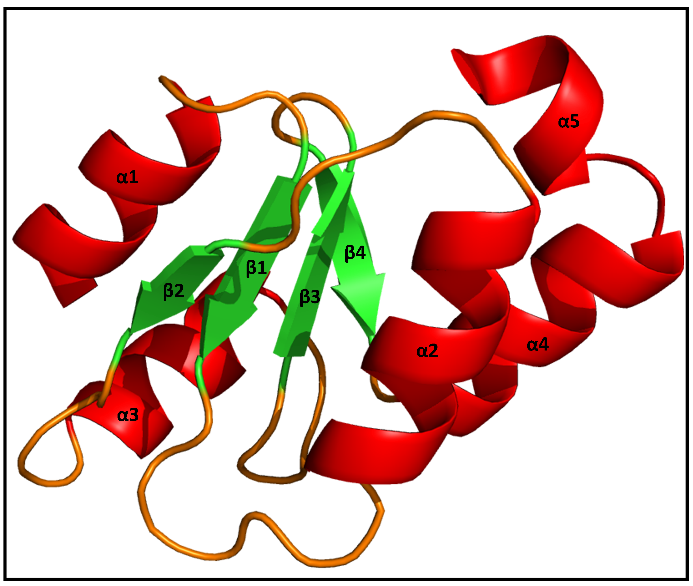


**Supplementary Figure S7**. Modeling of the Grx domain of BmTrxR. Sequence of the open reading frame of isoform d. Here, the secondary structure is shown only for the Grx domain (1-102). Cysteines of this domain are highlighted in yellow. The WP motif is highlighted in magenta.


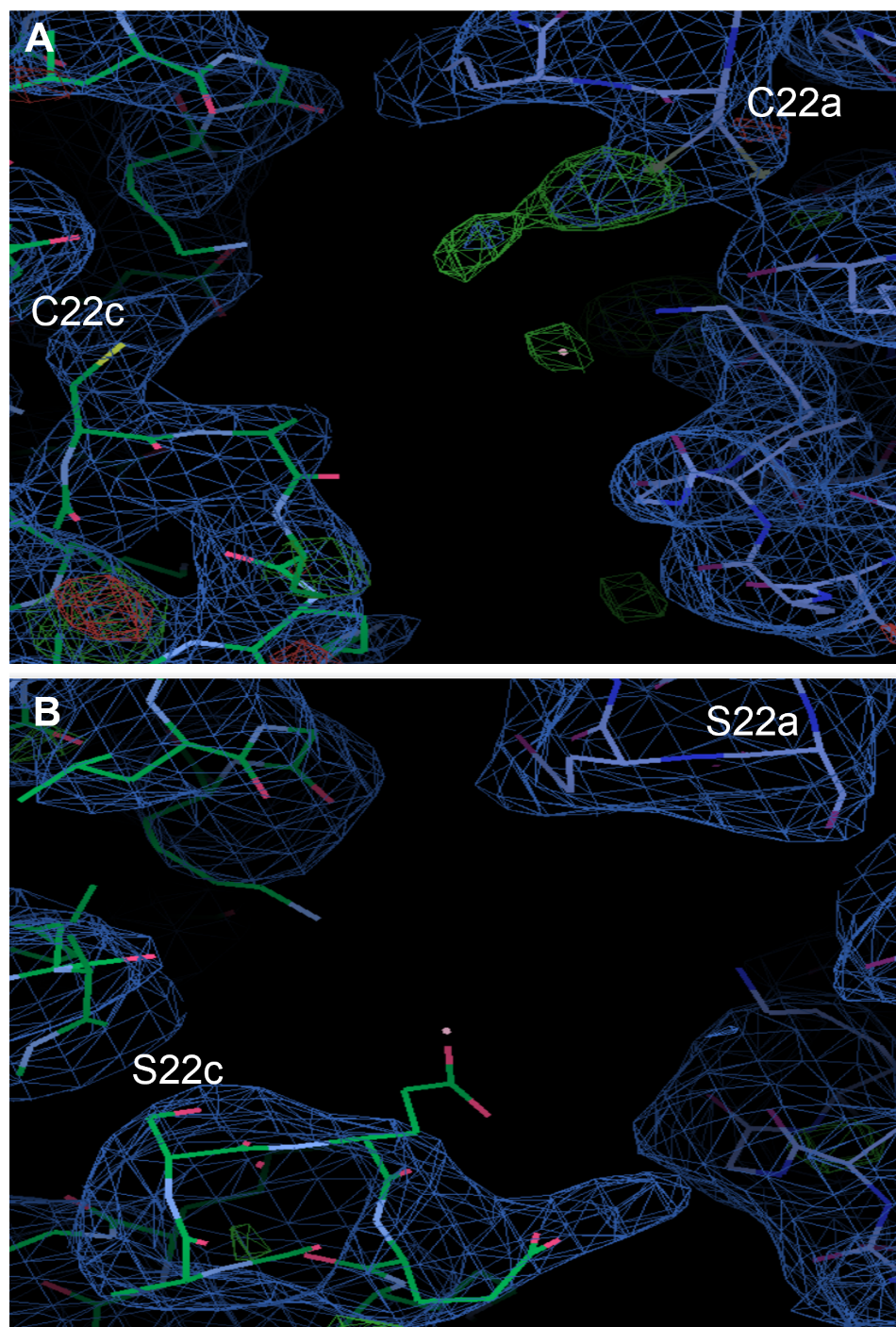


**Supplementary Figure S8.** Comparison of the electron density map between C22 of subunit “a” and “c” of the WT,isoform B BmTrxR (R=3 Å; panel A), characterized by a selenocysteine content of about 15-20%, and around S22 of the isoform D BmTxrR-C22S mutant (100% Sec incorporation; R=3.7 Å; panel B).


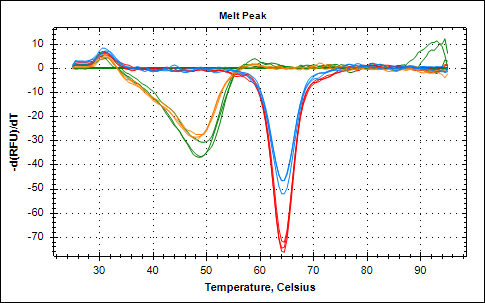


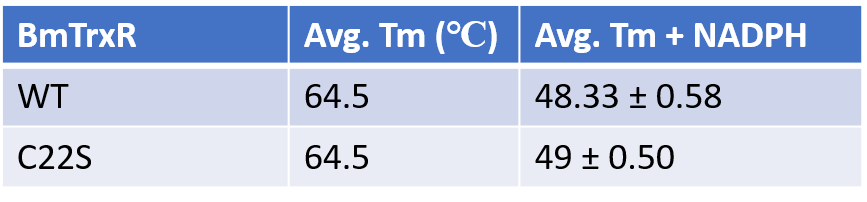


**Supplementary Figure S9.** Derivative graphs of ThermoFAD with BmTrxR isoform D and C22S (both at 3.8 µM) with and without 1 mM NADPH. Curves are WT alone – blue; C22S alone – red; WT + NADPH – orange; C22S + NADPH – green. The average melting temperatures are shown in the table.

Trichinella_pseudospiralis. ----------------------------MSAALNHRIYR------MKDSSMPPV------ 20

Brugia_malayi -------------------------------------------------M-----SPIPN 6

Ascaris_suum MLGTALLLRDLAGYVCHLCCQPTTLQYFQGRAVKEIKAEASEESAGVEDP-----SMAPT 55

Caenorhabditis_elegans MKS----LTELFGCF-----KRQPRQQEASSPANPHVSDTL-SMGVAASGMPPPKRPAPA 50

Haemonchus_contortus -------------------------------------------MGAVASAMPAARRRDEK 17

Necator_americanus -------------------------------------------MGAVAASMPNS------ 11

Angiostrongylus_cantonensis ---------------------------------------MG-GVGAVMRSSAGS------ 14

Trichinella_pseudospiralis --------DNVK-EPENFGSLLELIVNSKTIAVVNGHRIE-----LNKINDYLLS--VDS 64

Brugia_malayi RVSS----------GSLADAVFKSACEERILLAYADYNPD-----MTKVVNLFSKYNE-- 49

Ascaris_suum ASPG----------ASAAERVFKEASEKRTVVVYTERDAE-----LQKITDILTNRNE-- 98

Caenorhabditis_elegans ESPTLPGETLVDAPGIPLKEALKEAANSKIVIFYNSSDEEKQLVEFETYLNSLKEPADAE 110

Haemonchus_contortus SS-SSPDRTR-AKLDENAESILHTVATSKVAIIYSGYES-----NIQKYLSIIKSCESDE 70

Necator_americanus ---SSSRARR-AETHVNVSSILEAISTAKLTIVHSGEES-----EVNACLNVIKTCGYAG 62

Angiostrongylus_cantonensis ---QLSRIRR-LETQVNVSSVLNAIASSKVVVVHSGDSA-----VLKSYADIVENSGYKE 65

:. : . . .

Trichinella_pseudospiralis KPCQIADIKSISDRDRVISNLFDISGRNELPLIFVKGDCVGGVEDLKKLVENGVLKEWLA 124

Brugia_malayi ------TVNTVRVSNDAVKDILEIVGWPSMPLIFVKGNCCGGFKELYQLEESGFLNEWLK 103

Ascaris_suum ------TVELLKISHVAAKIVMNIVQRTTMPLIFIKGDCVGGLADLLKLEESGALNEWLK 152

Caenorhabditis_elegans KPLEIPEIKKLQVSRASQKVIQYLTLHTSWPLMYIKGNAVGGLKELKALKQ-DYLKEWLR 169

Haemonchus_contortus NHI---DVVRLCVNDKVKDRIQLITLRNQWPLIFIKGDAVGGLDELRKLADTKVLPEWLK 127

Necator_americanus -NV---EVTRLRVNGPAKDRLQLITLRNQWPLVFVKGDAVGGVEELKKLANKRILAEWLK 118

Angiostrongylus_cantonensis -NV---DVVQIRVNDSGKDRLQFITLRNEWPLVFIKGDAVGGLDELKMLSETGVLAEWLK 121

: : . : : **:::**:. **. :* * : * ***

Trichinella_pseudospiralis DHQYDLVVIGGGSGGLAAAKDAAVAGKRVAVLDFVTPTPLGTTWGSLGGTCVNVGCIPKK 184

Brugia_malayi EHEYDLAIVGGGSGGLAAAKEAVRLGKKVVCLDFVKPSAMGTTWG-LGGTCVNVGCIPKK 162

Ascaris_suum EHNYDLVVIGGGSGGLAAAKEAARLGKKVLCLDFVKPSVMGTTWG-LGGTCVNVGCIPKK 211

Caenorhabditis_elegans DHTYDLIVIGGGSGGLAAAKEASRLGKKVACLDFVKPSPQGTSWG-LGGTCVNVGCIPKK 228

Haemonchus_contortus DHNYDLIVVGGGSGGLAAAKMAALHGKKVAVLDFVKPSPQGSTWG-LGGTCVNVGCIPKK 186

Necator_americanus DHQYDLIVIGGGSGGLAAAKMAASHGKKVAVLDFVKPSPQGSTWG-LGGTCVNVGCIPKK 177

Angiostrongylus_cantonensis DHQYDLIVIGGGSGGLAAAKMAAGFGKKIAVLDYVKPSPQGTSWG-LGGTCVNVGCIPKK 180

:* *** ::*********** * **:: **:*.*: *::** **************

Trichinella_pseudospiralis LMHCSSLLGKNLADARNFGWKYGD-EVKHSWTDMVTAVQSHITALNWNYRVQLREKAVTY 243

Brugia_malayi LMHQAALLGEYIEDAKKFGWEIPEGAIKLNWHQLKNAVQNHIASLNWGYRVQLKEKSVTY 222

Ascaris_suum LMHQAALLGEYIGDAKKFGWEIPKGDMKLNWEKMRNAIQDHIASLNWGYRVQLRERSVTY 271

Caenorhabditis_elegans LMHQASLLGHSIHDAKKYGWKLPEGKVEHQWNHLRDSVQDHIASLNWGYRVQLREKTVTY 288

Haemonchus_contortus LMHQAAILGHSIKDAKMFGWKIPEGEINHNWENLRNAVQDHISSLNWGYRVQLREKQVTY 246

Necator_americanus LMHQASLLGHSIKDAKMFGWKLPEGDVTHNWKNLRDGVQDHIASLNWGYRVQLREREVTY 237

Angiostrongylus_cantonensis LMHQASLLGHSIKDAKMFGWKIPDGEIAHNWMTLKNGVQDHIAALNWGYRVQLREKQVTY 240

*** :::**. : **: :**: . : .* : .:*.**::***.*****:*: ***

Trichinella_pseudospiralis LNAFGTFVGSHSIKATDKRKKEQIITSDRFLIATGLRPRYLD-VPGVKEYCITSDDIFSL 302

Brugia_malayi MNSYATFTGSHELSVKNKKGKVEKVTADRFLIAVGLRPRFPD-VPGALECCISSDDLFSL 281

Ascaris_suum SNAYGVFTGSHELTTTNKKKKVEKVTADRFIIATGLRPRYPD-VPGAKECCISSDDLFSL 330

Caenorhabditis_elegans INSYGEFTGPFEISATNKKKKVEKLTADRFLISTGLRPKYPE-IPGVKEYTITSDDLFQL 347

Haemonchus_contortus INSYGRFTGPFEISATNKKGEVEKLTADRFLIATGLRPRYPPDVPGVREYCVTSDDLFSL 306

Necator_americanus INSYGVFTGPFEITATNKKGVSEKITADRFLIATGLRPRYPENTPGAREYTITSDDLFSL 297

Angiostrongylus_cantonensis INSYGKVTGPFEISATNKKGAVEKLTADRFLIATGLRPRYPENVPGAREYCITSDDLFSL 300

*::. ..* ..:...:*: : :*:***:*:.****:: **. * ::***:*.*

Trichinella_pseudospiralis PYCPGKTLCVGASYVSLECAGFLRGLGLDVTVMVRSILLRGFDQDMANRIGNHMQTVEGV 362

Brugia_malayi PYNPGKTLCVGASYVSLECAGFLKGIGNDVTVMVRSVLLRGFDQDMAERIKKHMTER-GV 340

Ascaris_suum TYNPGKTLCVGASYVSLECAGFLKGIGNDVTVMVRSILLRGFDQDMAERIRRHMMTH-EI 389

Caenorhabditis_elegans PYSPGKTLCVGASYVSLECAGFLHGFGFDVTVMVRSILLRGFDQDMAERIRKHMIAY-GM 406

Haemonchus_contortus PYPPGKTLCVGASYVSLECAGFLQGLGYDVTVMVRSILLRGFDQDMAERIRAHMKEC-GV 365

Necator_americanus PYPPGKTLCVGASYVSLECAGFLKGLGFDVTVMVRSILLRGFDQDMAERIRRQMLDY-GV 356

Angiostrongylus_cantonensis PYAPGKTLCIGASYVSLECAGFLKGLGFDVTVMVRSILLRGFDQDMAERIKTHMKEY-GV 359

* ******:*************:*:* ********:**********:** :* :

Trichinella_pseudospiralis RFIYQCIPTKIERFK---DGQPGLLRVTAKKEDGE-----EVVDEYNTVLIAIGRDALTD 414

Brugia_malayi KF-VQCVPIKYERLKKPTDSEPGMIRVHTMQEDED-GTK-EVTEDFNTVLMAIGRDAMTD 397

Ascaris_suum KF-INAVPTKYERIEEPADDKPGLVRVYWEETSQD-GEKTPCTQDFNTVLMAIGRDAVTD 447

Caenorhabditis_elegans KF-EAGVPTRIEQIDEKTDEKAGKYRVFWPKKNEETGEMQEVSEEYNTILMAIGREAVTD 465

Haemonchus_contortus KF-ENAVPTRIEEIEPKTKKQAGRLRVFFARKISD-TETEEHSEEFNTVVIAIGRDAMTK 423

Necator_americanus KF-VGAVPSRIEELEPKTKTKAGKLRVYWQEAHED-GTKVESSDDFDTVVMAIGRDAMTN 414

Angiostrongylus_cantonensis KF-ESAVPSRIEEIEPKTKNNAGRLRVFWNRTFAD-GSKAETSEEFNTVVIAIGRDAMTH 417

:* :* : *.:. . : * ** . : ::::*:::****:*:*.

Trichinella_pseudospiralis ALNLDKVGVQTNAKNKKIVCYSNEQSCTAPYIYAIGDVLDGGLELTPVAIKAGRLLVKRL 474

Brugia_malayi DLGLDVVGVNRAKSGK--IIGRREQSVSCPYVYAIGDVLYGSPELTPVAIQAGKVLMRRL 455

Ascaris_suum EMGLELVGVERTKSGK--IVGRREQS-TCPYVYGIGDVLNGCPELTPVAIQAGRVLMRRL 504

Caenorhabditis_elegans DVGLTTIGVERAKSKK--VLGRREQSTTIPWVYAIGDVLEGTPELTPVAIQAGRVLMRRI 523

Haemonchus_contortus DIGLDVVGVETASNGK--VKGRREQSLTCPYVYAIGDVLANTPELTPVAIQAGKVLMNRL 481

Necator_americanus DIGLNIVGVETNRAGK--VKGRREQSLTCPYVYALGDVLEGTPELTPVAIQAGKVLMKRL 472

Angiostrongylus_cantonensis DIGLDKLRVETDNRGK--VKGRREQSLTCPYVYAIGDVLENTPELTPVAIQAGRVLMRRL 475

:.* : *: * : .*** : *::*.:**** . *******:**::*:.*:

Trichinella_pseudospiralis FGLSSNLCEYHLVPTTVFTPLEYGCCGLSEEKAIEKYGENNIEVFHSYFTPLEYTVPKRG 534

Brugia_malayi FTGSSELTEYDKIPTTVFTPLEYGSCGLSEYSAIQKYGKENINVYHNVFIPLEYAVTERK 515

Ascaris_suum ITGNSELTEYDQVPTTVFTPLEYGCCGLAEEAAIQKYGKENINVYHNVFIPLEYSVPERV 564

Caenorhabditis_elegans FDGANELTEYDQIPTTVFTPLEYGCCGLSEEDAMMKYGKDNIIIYHNVFNPLEYTISERM 583

Haemonchus_contortus YYGSDLLTEYDEVPTTVFTPLEYGCCGLTEENAKQRYGEDNVIVYHAVFIPLEYTVAERM 541

Necator_americanus YGGADDLTEYNDVPTTVFTPLEYGCCGLSEENAYAHYGKDNVIVYHAVFFPLEYTVAERA 532

Angiostrongylus_cantonensis YDGVDELTEYDNVPTTVFTPLEYGCCGLSEESAYERFGKENVIVYHAVFFPLEYTISERM 535

. * **. :***********.***:* * ::*::*: ::* * ****:: :*

Trichinella_pseudospiralis DSEHCYAKLICNKQDDMRILGFHLLGPNAGEITQGFAVGLKLKATKHDFDQLVGIHPTCA 594

Brugia_malayi EKTHCYCKLICLKNEQDLILGFHILTPNAGEITQGFAIALKFDAKKADFDRLIGIHPTVA 575

Ascaris_suum ENSHCYCKLICLKTEQDLVLGYHILAPNAGEITQGFAIGLKLKAKKADFDRLIGIHPTVA 624

Caenorhabditis_elegans DKDHCYLKMICLRNEEEKVVGFHILTPNAGEVTQGFGIALKLAAKKADFDRLIGIHPTVA 643

Haemonchus_contortus DKDHCYCKLICLASDNERVIGFHILAPNAGEITQGFGIALKLGGTKADFDRLIGIHPTVA 601

Necator_americanus EKDHCYCKLICLKNEKERVVGFHILSPNAGEITQGFGIALKLGGTKADFDRLIGIHPTVA 592

Angiostrongylus_cantonensis DKDHCYCKLICLKNEEERVLGFHILAPSAGEITQGFGIALKLGARKSDFDRLIGIHPTIA 595

:. *** *:** :. ::*:*:* *.***:****.:.**: . * ***:*:***** *

Trichinella_pseudospiralis EVFTQLTVTKSS-QQELKKTGCUG 617

Brugia_malayi ENFTTLTLVKEDG-QTLKATGCUG 598

Ascaris_suum ENLTTLTLVKEEG-QQLKASGCUG 647

Caenorhabditis_elegans ENFTTLTLEKKEGDEELQASGCUG 667

Haemonchus_contortus ESFTTLFLVKVPGGEELKATGCUG 625

Necator_americanus ESFTTLYLVKKEGEDELKATGCUG 616

Angiostrongylus_cantonensis ESFTTLSLIKKEGEAELKATGCUG 619

* :* * : * *: :****

**Supplementary Figure S10.** Comparison of TrxR sequences from nematodes. Representative sequences of nematode TrxRs from different clades (International Helminth Genomes Consortium. Comparative genomics of the major parasitic worms. Nat Genet. 2019, 51, 163-174) were obtained from GeneBank and aligned using Clustal Omega (Madeira et al. The EMBL-EBI search and sequence analysis tools APIs in 2019. Nucleic Acids Res. 2019, 47, W636-W641): *Trichinella* *pseudospiralis* from clade I, *Brugia* *malayi* from clade IIIc (isoform d), *Ascaris* *suum* from clade IIIb, *Caenorhabditis* *elegans* from clade V other, *Haemonchus* *contortus* from clade Va, *Necator* *americanus* from clade Vc, and *Angiostrongylus* *cantonensis* from clade Vb. The Grx domains are shown in blue, the TrxR domains in black. The TrxR domains are more highly conserved than the Grx domains across all clades.

a. Clade IIIc

Thelazia_callipaeda MPPIDGVLNDGVTADAVFKSACEERALIAYTQYSNDITEALALFSKYDVVVKAVRVSDCT 60

Onchocerca_ochengi MPPT-YPVCDRTAADAVFKSACEERVLLAYTNCDSDITKIVNLFSKYGETVHKVRVNHDA 59

Onchocerca_flexuosa MPPT-HPICYRTAADAVFKSACEERVLLAYTNCDSDVTKIVNLFSKYGETVHKVHVNHDA 59

Acanthocheilonema_viteae MSPSSNR-SNEAAADAVFKSACKERVLLAYTDYNLDITKIVNLFSKYGERVNTVRVSNDA 59

Litomosoides_sigmodontis MPPTSNAVSSGAAADAVFKSACKERVLLAYIDYNLDITKIVNLFSKYGETVSTVRVSRDA 60

Loa_loa MPPTSNLVISGAAASAVFKSACEERVLLAYTDYNSDITKVVNLFSKYGETVNTVRVSNDA 60

Wuchereria_bancrofti MSPISNRASSGASADAVLKSACEERILLAYADYNPDITKVMNLFSKYNETVNTVRVSNDA 60

Brugia_malayi MSPIPNRVSSGSLADAVFKSACEERILLAYADYNPDMTKVVNLFSKYNETVNTVRVSNDA 60

Brugia_pahangi MSPISNRVSSGSLADAVFKSACEERILLAYADYNPDMTKVVNLFSKYNETVNTVRVSNDA 60

* * *.**:****:** *:** : . *:*: : *****. * *:*. :

Dracunculus medinensis -------------------------------MF---NQFILRHFSKRQLKSFSLNISQKD 26

Thelazia_callipaeda ANTILEIVEWPSMPLVFIKGDCCGSLKELHHHEAKGTLSEWLKEHQYDLAVI 112

Onchocerca_ochengi ANHMLEIVGWSSMPLIFVKGNCCGGFKELYQLEKNGSLSEWLKEHQYDLAVI 111

Onchocerca_flexuosa ANHMLEIVGWPSMPLIFVKGNCCGGFKELYQLEKNGSLSEWLKEHQYDLAVI 111

Acanthocheilonema_viteae AKNILEIVRWPSMPLIFVKGDCCGGLKELHQLEENGSLNEWLKEHQYDIAVV 111

Litomosoides_sigmodontis VKDILEIVKWPSMPLIFVKGDCCGGLREIHQLEENGFLNEWLKEHQYDLAVV 112

Loa_loa VKSVLEIVGWPSMPLIFVKGNCCGSFKELYQLEENGSLNEWLKEHQYDLAVV 112

Wuchereria_bancrofti VKNILEIVGWPSMPIIFVKGNCCGGFKELYQLEESGSLNEWLKEHQYDLAVV 112

Brugia_malayi VKDILEIVGWPSMPLIFVKGNCCGGFKELYQLEESGFLNEWLKEHEYDLAIV 112

Brugia_pahangi VKNILEIVRWPSMPLIFVKGNCCGGFKELYQLEESGFLNEWLKEHEYDLAIV 112

.: :**** * ***::*:**:***.::*::: * .* *.******:**:*::

Dracunculus medinensis. AKIMLNIVKHNNMPLIFIKGDCVGGLAELLALDQKGVLDSWLAKHDYDLAVI 78

b. Other Clade V

Caenorhabditis_brenneri MKSFTNLFGCFKRNPRQE-AAAPADRSTAGASSMGAVASGMPPPKRPAPTDSPGPPVENN 59

Caenorhabditis_elegans ---------------------------------MGVAASGMPPPKRPAPAESPTLPGETL 27

Caenorhabditis_briggsae MKSLTDLFGCFKRNPRRGDEAASANQSTSDSPPMGAVASGMPPPKRRAPTETKTPPGERN 60

Caenorhabditis_latens MKSLGELFGCFKRQPRQGDATAPADQSTSGTPSMGAVASGMPPPKRPAPAESPTLSDERN 60

Caenorhabditis_remanei MKSLGELFGCFKRQPRQGDATAPANQSTSDTPSMGAVASGMPPPKRPAPAESPTLPDERN 60

**..********* **::: *

Caenorhabditis_brenneri VDAPGTPLKDALKQANNAKVAVFFTG--TEEEKQIHEIQAILNALKDDPNVENPVEVPEI 117

Caenorhabditis_elegans VDAPGIPLKEALKEAANSKIVIFYNS--SDEEKQLVEFETYLNSLKEPADAEKPLEIPEI 85

Caenorhabditis_briggsae VDDTGIPLKEALKQANDAKFAVFCSGNNEEEEKQISEIESILKTLKDPPETEKPLEIPEI 120

Caenorhabditis_latens VDEPGIPLKEALKEANNAKIAVFYNK--ADEEKQILEIEAILKGLKDPSDVEKPLEIPDV 118

Caenorhabditis_remanei VDEPGIPLKEALKEANNAKIAVFYSHNTFDEEKQILEIEAILKALKDRADVEKSLEIPDV 120

** * ***:***:* ::*..:* . :****: *::: *: **: :.*: :*:*::

Caenorhabditis_brenneri QKIQVSGFSKKAIQHLTLHNSWPLIYIKGNAVGGLKELQALKQDYLKEWLRDHTYDLIVI 177

Caenorhabditis_elegans KKLQVSRASQKVIQYLTLHTSWPLMYIKGNAVGGLKELKALKQDYLKEWLRDHTYDLIVI 145

Caenorhabditis_briggsae QRIQVSSSSKKAIQYLTLHDSWPLIYIKGNAVGGLKELKALKQDYLKEWLRDHTYDLIVI 180

Caenorhabditis_latens QRIRVSSASKKAIQYLTLHNSWPLIYIKGNAVGGLKELQALKKDYLKEWLRDHTYDLIVI 178

Caenorhabditis_remanei QRIRVSSASKKAIQYLTLHNSWPLIYIKGNAVGGLKELQALKKDYLKEWLRDHTYDLIVI 180

::::** *:*.**:**** ****:*************:***:*****************

c. Clade I

Trichuris_trichiura ------------------------------------MLQDAFF----------------- 7

Trichinella_papuae LISKHCYSRMLTFTVRANRDSSMPPVDNVKEPEDFSSLLELIVNSKTIAVVNGHRIELNK 60

Trichinella_spiralis ----------------------MPPVDNVKKPQNLGSLLELIFNSKTITVVSGRRLKLNK 38

* : :.

Trichuris_trichiura -KNVILRI-----TFTFEFYCLERESIVKSLLSYAGREQLPLVFVKGDCVGCACDLEELN 61

Trichinella_papuae INDYLLSVDSKPCQIADIKSISDRDRVISNLFDISGRKELPLIFVKGDCVGGVEDLRKLV 120

Trichinella_spiralis INDYLLSIGSKPCQIADISSIADKDRVLRYLFDISCRNLLPLIFVNGDCVGGVEELARLV 98

:: :* : :: ::: :: *:. : *: ***:**:***** . :* .*

Trichuris_trichiura KRGLLKECLEDHKYDLVVV 80

Trichinella_papuae ENGVLKEWLADHQYDLVVI 139

Trichinella_spiralis EKGVLKEWLADHQYDLVVI 117

:.*:*** * **:*****:

d. Clades Va, Vb, Vc

Necator_americanus -------------------------------------------------------MGAVA 5

Ancylostoma_caninum -------------------------------------------------------MGAVA 5

Ancylostoma_ceylanicum MKEALKIITCLQRYRNCALSTLCALSTGASLSTRFTLNSALHTSVCRVAAVSAPGMGAVA 60

Angiostrongylus_costaricensis ------------------------------------------------------------ 0

Dictyocaulus_viviparus -------------------------------------------------------MGGVA 5

Nippostrongylus_brasiliensis ------------------------------------------------------------ 0

Haemonchus_contortus -------------------------------------------------------MGAVA 5

Haemonchus_placei -------------------------------------------------------MGAVA 5

Heligmosomoides_polygyrus ------------------------------------------------------------ 0

Necator_americanus ASMPNSSS--------SRARRAETHVNVSSILEAISTAKLTIVHSGEESEVNACLNVIKT 57

Ancylostoma_caninum ATMPNSDGSH-----YSRARRVETHVNVSSILEAISTAKLAIVHSGEESDVNACLNAIKT 60

Ancylostoma_ceylanicum ATMPNSTGSH-----YSRARRVETHVNVSSILEAISSAKLAIVHSGEESDVNACLNAIKT 115

Angiostrongylus_costaricensis -----------------------------------------------------YADIIEN 7

Dictyocaulus_viviparus AGMPSSAHSR-----QSRARRLETNVDVSSVLQTISSSKVAIIHSGESTQLKNCTEVIRT 60

Nippostrongylus_brasiliensis -------------------------------MHPWA----------ASTNRRIYTNIFKE 19

Haemonchus_contortus SAMPAARRRDEKSSSSPDRTRAKLDENAESILHTVATSKVAILYSGYESNIQKYLSIIKS 65

Haemonchus_placei SAMPAARRRDENSSSSPDRTRAKLDENAESILHTVATSKVAIVYSGYESNIQKYLSIIKS 65

Heligmosomoides_polygyrus ----------------------------------------------MSLSHFQYLDVLEQ 14

. :.

Necator_americanus CGYA-GNVEVTRLRVNGPAKDRLQLITLRNQWPLVFVKGDAVGGVEELKKLANKRILAEW 116

Ancylostoma_caninum CGYA-GNVEVTRLRVNEPAKDRLQLITLRNQWPLVFVKGDAVGGVSELKKLAENRVLAEW 119

Ancylostoma_ceylanicum CGYA-GNVEVTRLRVNEPAKDRLQLITLRNEWPLVFVKGDAVGGVSELKKLADSRILCEW 174

Angiostrongylus_costaricensis SGYT-ENVEVVQIRVNDSGKDRLQFITLRNEWPLVFIKGDAVGGLDELKKLSETGVLSEW 66

Dictyocaulus_viviparus CGYE-ENVEVVQIRVNESGKERLQFITLRNQWPLIFIKGDAIGGVDELKKLSDAKILGEW 119

Nippostrongylus_brasiliensis --NDAAEDDVTRVRVSDAVKERLQFLTLRNQWPLVFIKGDAVGGLDELKSVAETGLLKEW 77

Haemonchus_contortus CESDENHIDVVRLCVNDKVKDRIQLITLRNQWPLIFIKGDAVGGLDELRKLADTKVLPEW 125

Haemonchus_placei CESDENHIDVVRLCVNDKVKDRIQLITLRNQWPLIFIKGDAVGGLDELRKLADTKVLPEW 125

Heligmosomoides_polygyrus CD--AKHEDVVQLHVNDSVKERIQFVTLRNQWPLVFIKGDAVGGLDELKQLADTKVLPEW 72

. :*.:: *. *:*:*::****:***:*:****:**:.**:.::: :* **

Necator_americanus LKDHQYDLIVI 127

Ancylostoma_caninum LKDHQYDLIVI 130

Ancylostoma_ceylanicum LKDHQYDLIVV 185

Angiostrongylus_costaricensis LKDHQYDLIVI 77

Dictyocaulus_viviparus LRNHQYDLVVI 130

Nippostrongylus_brasiliensis IKDHQYDLIVI 88

Haemonchus_contortus LKDHNYDLIVV 136

Haemonchus_placei LKDHTYDLIVV 136

Heligmosomoides_polygyrus LKDHQYDLIVI 83

:::* ***:*:

e. Clade IIIb

Toxocara_canis MAPTTSPGSSAAERVFKEASEKRAVVVYTDRDAELQKITDMLTKRNETVELLKISRVAAKNVLNVVQRD 69

Ascaris_suum MAPTASPGASAAERVFKEASEKRTVVVYTERDAELQKITDILTNRNETVELLKISHVAAKIVMNIVQRT 69

****:***:**************:*****:**********:**:***********:**** *:*:***

Toxocara_canis NVPLIFIKGDCVGGLVDLLKLEESGALDEWLKQHKYDIVVV 110

Ascaris_suum TMPLIFIKGDCVGGLADLLKLEESGALNEWLKEHNYDLVVI 161

.:*************.***********:****:*:**:**:

**Supplementary Figure S11.** Comparison of the N-terminal extensions from TrxR proteins grouped by clade. Sequences of nematode TrxRs were obtained from GeneBank, the N-terminal extensions were extracted and were aligned using Clustal Omega (Madeira et al. The EMBL-EBI search and sequence analysis tools APIs in 2019. Nucleic Acids Res. 2019, 47, W636-W641). Grouping of nematodes into clades is as described (International Helminth Genomes Consortium. Comparative genomics of the major parasitic worms. Nat Genet. 2019, 51, 163-174). Cysteine residues are highlighted in yellow except for Cys22 in BmTrxR and related sequences that are highlighted in green. The sequence of the Grx domain of BmTrxR is the only one in cyan.

|  | **BmTrxR-NADPH** | **BmTrxR_APO** | **BmTrxR-Auranofin** |
| --- | --- | --- | --- |
| PDB IDs  **DATA REDUCTION STATISTICS** | 7PUT | 7P0X | 7PVJ |
| Space group | C2 2 21 | C2 2 21 | C2 2 21 |
| Unit cell dimensions (Å) | 147.39 260.29 130.33 | 146.92 258.98 129.07 | 147.33 261.66 130.29 |
| Resolution range (Å) | 49.13-2.80 (2.87-2.80)* | 48.90-2.55 (2.60-2.55)* | 49.31-3.10 (3.21-3.10)* |
| mean I/sigma(I) | 22.2 (3.1) | 28.8 (2.3) | 18.6 (3.1) |
| Completeness (%) | 99.9 (100) | 100 (100) | 100 (100) |
| CC1/2 (%) | 99.9 (87.4) | 100 (80) | 99.8 (90.4) |
| Rmerge (%) | 4.6 (56.4) | 3.2 (81.9) | 8.3 (68.8) |
| Number of unique reflections | 61904 (4537) | 80241 (4505) | 46008 (4474) |
| Redundancy | 7.2 (7.6) | 6.6 (6.8) | 12.8 (13.6) |
| **REFINEMENT STATISTICS**  Resolution range (Å) | 49.13-2.80 (2.87-2.80)* | 48.90-2.55 (2.62-2.55)* | 49.36-3.10 (3.18-3.1)* |
| No. reflections | 58828 (4278)* | 76369 (5556) | 43675 (3159) |
| R/Rfree | 0.20/0.23 (0.30/0.39)* | 0.20/0.24 (0.32/0.33) | 0.19/0.23 (0.26/0.28) |
| Mean B-factor (Å) | 103.5 | 94.4 | 117.5 |
| RMS bond lengths deviations | 0.010 | 0.009 | 0.008 |
| RMS bond angles deviation | 1.62 | 1.56 | 1.57 |
| **Ramachandran (%)**  Favored | 95 | 95 | 95 |
| Allowed | 4.7 | 4.7 | 5 |
| Disallowed | 0.3 | 0.3 | 0 |

*In parenthesis the values for the OuterShell

**Supplementary Table S1:** summary of X-ray data reduction and structural refinement statistics
